# Systematic identification of CRISPR off-target effects by CROss-seq

**DOI:** 10.1093/procel/pwac018

**Published:** 2022-07-15

**Authors:** Yan Li, Shengyao Zhi, Tong Wu, Hong-Xuan Chen, Rui Kang, Dong-Zhao Ma, Zhou Songyang, Chuan He, Puping Liang, Guan-Zheng Luo

**Affiliations:** MOE Key Laboratory of Gene Function and Regulation, Guangdong Province Key Laboratory of Pharmaceutical Functional Genes, State Key Laboratory of Biocontrol, School of Life Sciences, Sun Yat-sen University, Guangzhou 510275, China; MOE Key Laboratory of Gene Function and Regulation, Guangdong Province Key Laboratory of Pharmaceutical Functional Genes, State Key Laboratory of Biocontrol, School of Life Sciences, Sun Yat-sen University, Guangzhou 510275, China; Department of Chemistry, University of Chicago, Chicago, IL 60637, USA; MOE Key Laboratory of Gene Function and Regulation, Guangdong Province Key Laboratory of Pharmaceutical Functional Genes, State Key Laboratory of Biocontrol, School of Life Sciences, Sun Yat-sen University, Guangzhou 510275, China; MOE Key Laboratory of Gene Function and Regulation, Guangdong Province Key Laboratory of Pharmaceutical Functional Genes, State Key Laboratory of Biocontrol, School of Life Sciences, Sun Yat-sen University, Guangzhou 510275, China; MOE Key Laboratory of Gene Function and Regulation, Guangdong Province Key Laboratory of Pharmaceutical Functional Genes, State Key Laboratory of Biocontrol, School of Life Sciences, Sun Yat-sen University, Guangzhou 510275, China; MOE Key Laboratory of Gene Function and Regulation, Guangdong Province Key Laboratory of Pharmaceutical Functional Genes, State Key Laboratory of Biocontrol, School of Life Sciences, Sun Yat-sen University, Guangzhou 510275, China; Department of Chemistry, University of Chicago, Chicago, IL 60637, USA; Institute for Biophysical Dynamics, Department of Biochemistry and Molecular Biology, Howard Hughes Medical Institute, University of Chicago, Chicago, IL 60637, USA; MOE Key Laboratory of Gene Function and Regulation, Guangdong Province Key Laboratory of Pharmaceutical Functional Genes, State Key Laboratory of Biocontrol, School of Life Sciences, Sun Yat-sen University, Guangzhou 510275, China; MOE Key Laboratory of Gene Function and Regulation, Guangdong Province Key Laboratory of Pharmaceutical Functional Genes, State Key Laboratory of Biocontrol, School of Life Sciences, Sun Yat-sen University, Guangzhou 510275, China

Dear Editor,

The CRISPR-mediated genome editing tools, including nucleases, base editors (ABE/CBE), transposases/recombinases, and prime editor (PE), have been extensively applied in basic and clinical researches, although the off-target effect remains a major concern (Anzalone et al., 2020). Recently, various methods have been developed to assess the specificity and accuracy of different tools (Zhang et al., 2021), yet each method is designed for limited editing systems, and none of them can simultaneously detect off-target sites *in vivo* and *in vitro*. A versatile method for profiling genome-wide off-target effects of various tools remains lacking.

A common feature of CRISPR-mediated genome editors is their dependence on RNA-guided site-specific DNA recognition, where an R-loop structure is formed and the opposite single-stranded DNA (ssDNA) is exposed (Anzalone et al., 2020). The recently reported KAS-seq (kethoxal-assisted single-stranded DNA sequencing) provides a unique and facile approach to map ssDNA *in situ* (Weng et al., 2020; Wu et al., 2020). We thus speculated that the binding events of CRISPR-mediated genome editors could be revealed via detecting the ssDNA component of R-loop. To test this, we carried out CRISPR-mediated genome editing in cells or isolated genomic DNA. The N_3_-kethoxal labeled ssDNA region allows subsequent biotinylation through azido group-associated click reaction and can be enriched for deep sequencing (Fig. 1A). Meanwhile, an optimized labeling condition was developed for *in vitro* CRISPR-Cas targeting assay (Fig. S1). We termed this method CROss-seq (CRISPR Off-targeting ssDNA sequencing) and speculated that it could detect targeting specificity of multiple CRISPR-mediated genome editors and facilitate cross-validation of gRNA dependent off-target effects *in vivo* and *in vitro* (Fig. 1A).

**Figure 1.**
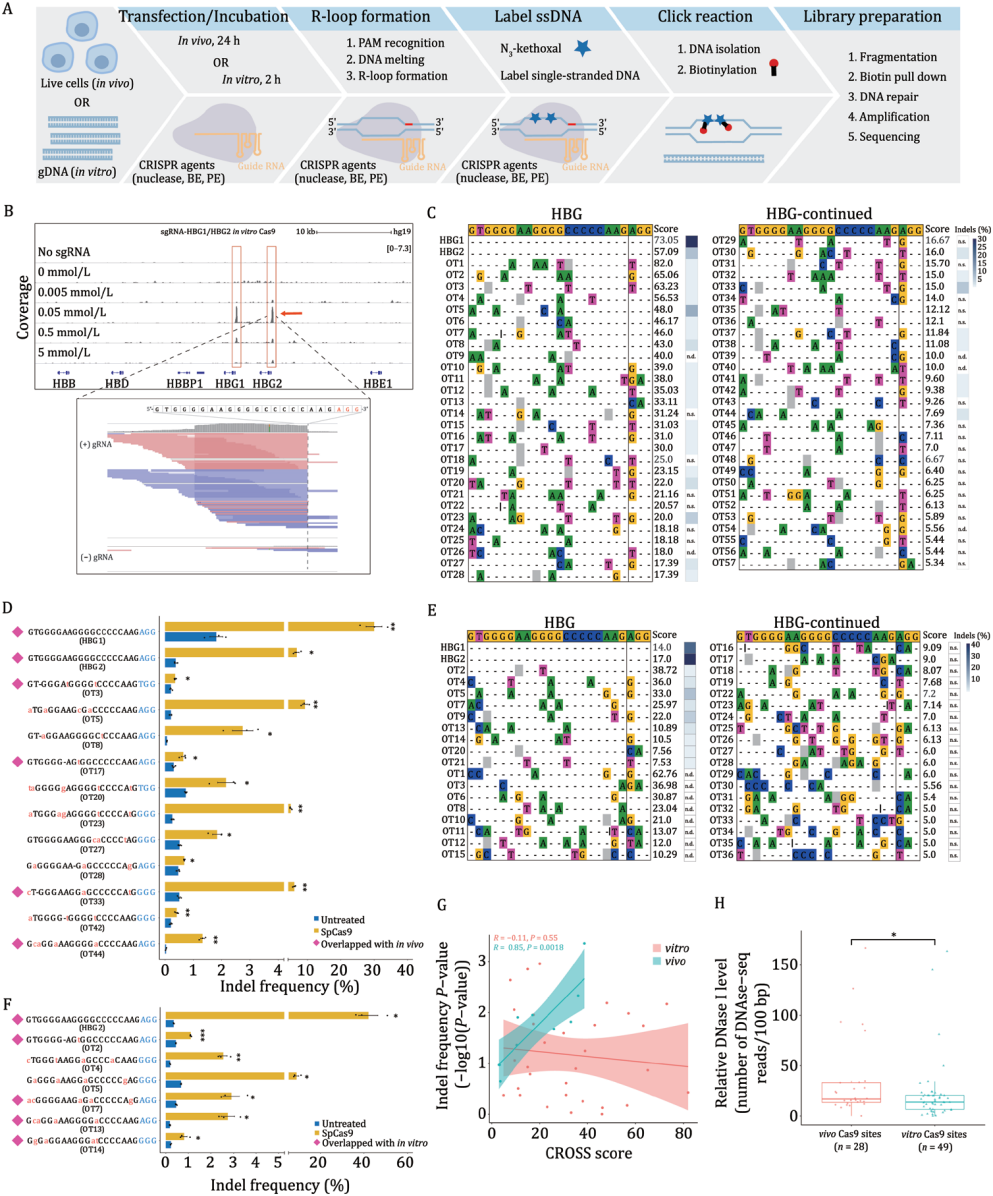
Genome-wide off-target sites induced by SpCas9 revealed by CROss-seq. (A) The schematics of CROss-seq. (B) The reads coverage from CROss-seq at the HBG target locus of the samples treated with different N_3_-kethoxal concentrations. Samples without sgRNA supplement were used as control. The *HBG1* and *HBG2* on-target sites are indicated by the red box. The zoomed-in window shows that most reads end in the SpCas9 cleavage site. (C) Off-target sequences and corresponding scores identified by SpCas9 CROss-seq *in vitro* (0.05 mmol/L N_3_-kethoxal). The indel frequencies for *HBG* locus in HEK293T cells calculated from the targeted deep sequencing result are shown on the right of each site. The on-target sequence is shown at the top of the alignment. Mismatched nucleotides are highlighted in color. Putative sgRNA bulges (gray) and target DNA bulges (black line) are shown. n.s., not selected for validation using targeted deep sequencing. n.d., not determined due to PCR failure. OT, off-target. (D) Off-target sites validated by targeted deep sequencing. Yellow and blue bars represent indel frequencies obtained from cells expressing *HBG* sgRNA or without sgRNA, respectively. Off-target sites overlapped with which identified *in vivo* are marked with a magenta diamond. PAM sequences are shown in blue. Mismatched bases are shown in red. OT, off-target. Statistical significance was calculated using a two-tailed unpaired *t*-test (*P < 0.05, **P < 0.01, ***P < 0.001). Error bars indicate s.e.m. (*n* = 3). (E) Off-target sequences and corresponding scores identified by SpCas9 CROss-seq *in vivo* (24-h group). The indel frequencies for *HBG* in HEK293T cells calculated from the targeted deep sequencing result are shown on the right of each site. The on-target sequence is shown at the top of the alignment. Mismatched nucleotides are highlighted in color. Putative sgRNA bulges (gray) and target DNA bulges (black line) are shown. n.s., not selected for validation using targeted deep sequencing. n.d., not determined due to PCR failure. OT, off-target. (F) Off-target sites validated by targeted deep sequencing. Yellow and blue bars represent indel frequencies obtained from cells expressing *HBG* sgRNA or without sgRNA, respectively. Off-target sites overlapped with which identified *in vitro* are marked with a magenta diamond. PAM sequences are shown in blue. Mismatched bases are shown in red. OT, off-target. Statistical significance was calculated using a two-tailed unpaired *t*-test (*P < 0.05, **P < 0.01, ***P < 0.001). Error bars indicate s.e.m. (*n* = 3). (G) Scatterplot of indel significance versus CROSS scores at sites captured by CROss-seq for CRISPR-Cas9 *in vivo* and *in vitro*. Pearson’s correlation was obtained with indel frequency *P*-value versus DNA cleavage scores at off-target sited identified by *in vivo* or *in vitro* CROss-seq. (H) DNase I hypersensitivity levels for 28 SpCas9 off-target sites *in vivo* (red) and 49 SpCas9 off-target sites *in vitro* (green). The statistical significance was calculated using a two-tailed unpaired *t*-test (*P < 0.05, **P < 0.01, ***P < 0.001).

First, we designed sgRNA for two target sites (*HBG1* and *HBG2*) (Liang et al., 2019) and conducted SpCas9 cleavage assay *in vitro*. To determine the optimal reaction condition, we used a gradient of N_3_-kethoxal working concentrations. Samples treated in parallel but without sgRNA were applied as negative controls. We found highly enriched signals at target sites in editing samples compared to the control sample (Fig. 1B). In addition, the enriched fragments showed a clear cleavage signal precisely at the putative editing site, confirming the effective identification of SpCas9 targeting (Fig. 1B).

Next, we defined a CROSS score that calculates the number of reads with cleavage signal at a given locus under normalized sequencing depth, and applied this score to evaluate the reliability (Fig. S2) (Kim et al., 2015). The optimized labeling condition yielded the largest number of putative off-target sites (Fig. S3A and S3B; Table S1). Consensus sequence analysis of these sites reflected the expected spacer sequence of the sgRNA, with better alignment in the PAM-proximal region (Fig. S3C). We then compared the candidate sites to the off-target sites reported by Digenome-seq using the same sgRNA (Liang et al., 2019). Among the 45 reported sites, 18 of them could be confirmed by CROss-seq (Fig. S3D). Notably, the Digenome-seq cleavage scores of the overlapped sites were significantly higher than the others (Fig. S3E). We tested other two sgRNAs (*HEK4* and *FANCF*) and compared to previous methods, showing the reproducibility of CROss-seq (Fig. S3F and S3G). To further validate the accuracy, we performed targeted deep sequencing on the selected top off-targets as well as two on-target sites according to CROSS scores (Fig. 1C). Indels were observed at 13 out of the 30 sites, with frequencies ranging from 0.28% to 20% (Fig. 1D). The use of another sgRNA targeting *HEK2* showed similar results (Fig. S4; Table S2).

We next applied CROss-seq *in vivo*. After 12- or 24-h Cas9 transfection, cells were treated with N_3_-kethoxal for 45 min. A negative control without sgRNA supplement was conducted in order to eliminate the background signals derived from endogenous ssDNA regions. The 24-h group showed strong CROss-seq signal intensity at the *HBG1* and *HBG2* target sites (Fig. S5A). Interestingly, reads around the targeting sites displayed both indels and truncations, suggesting that N_3_-kethoxal can effectively label the target region before and after the Cas9-induced DNA repair (Fig. S5A). The candidate off-target sites were then ranked and filtered by their CROSS scores (Fig. S2). Besides on-target sites, we identified three off-target sites from the 12-h group and 36 off-target sites from the 24-h group (Fig. S5B and S5C; Table S3), suggesting that the off-target effects positively correlated to the editing intensity in a time-dependent manner. Consensus sequence analysis of off-target sites recapitulated the designed spacer sequence, supporting the accuracy of CROss-seq in detecting sgRNA-dependent off-targets (Fig. S5D). CROss-seq using cells treated with sgRNA targeting another gene *HEK2* showed comparable results (Fig. S6; Table S4).

To evaluate the accuracy of CROss-seq *in vivo*, we selected the top off-target sites according to the CROSS scores and performed targeted deep sequencing. NGS data revealed that 10/11 (91%) of these sites showed an off-target cleavage signal *in cellulo* (Fig. 1E), and seven of them harbored obvious indels with frequency ranging from 1% to 40% (Fig. 1F). Compared to other cell-based and cell-free methods, CROss-seq showed the highest validation rate of off-targets (Fig. S5E).

*In vivo* and *in vitro* assays for off-target detection usually show widespread discrepancies (Kim and Kim 2018). CROss-seq offered the opportunity to evaluate the off-target effects *in vitro* and *in vivo* simultaneously. Through a comparative analysis, we found that *in vitro* CROss-seq assay revealed more off-target sites (Fig. S5F). To further evaluate their consistency, we found a strong correlation (*R* = 0.85) between CROSS score and indel level for the off-target sites detected *in vivo*. However, no such correlation was observed for *in vitro* assay (*R* = −0.11) (Fig. 1G). These results highlighted the importance of cross-validation of off-target effects through multiple strategies.

Compact chromatin structure hinders the nuclease access and thus results in fewer off-target sites (Kim and Kim 2018). We therefore compared the off-target sites to the DNase I hypersensitivity sites (DHSs), and found that sites identified by CROss-seq *in vivo* assay were more frequently overlapped with the DHSs (Fig. S5G). Moreover, the average DHSs signal intensity of these sites was significantly higher (Fig. 1H). These results collectively indicated the influences of chromatin accessibility in CRISPR-Cas9 genome editing, which again underlined the need for a cross-validation method applied simultaneously *in vitro* and *in vivo*.

The performance of CROss-seq in the SpCas9 system prompted us to apply it in base editor systems (Komor et al., 2016). After BE3 editing, the CROss-seq *in vitro* assay revealed an evident peak on the expected targeting locus compared to negative control (Fig. S7A). We also observed a clear truncation signature towards the cleavage site, reflecting the nickase activity of nCas9 in BE3 editing (Fig. S7A). Then we used the CROSS score for off-target sites detection and discovered 12 putative sites with a consensus sequence resembling the target sequence (Fig. S7B and S7C; Table S5). Notably, CROss-seq detected more off-target sites than Digenome-seq did (Kim et al., 2017), and only the on-target site was commonly detected by both methods (Fig. S7D). We then evaluated the off-target effects of the *in vivo* BE4max system (Koblan et al., 2018). We therefore adjusted the CROSS-scoring to assess the editing effects by calculating the ratio of reads with C-to-T or A-to-G conversion within the editing window (Fig. S8). As expected, we observed apparent CROss-seq peaks and C-to-T conversion at the *HEK2* target site (Fig. 2A; Table S6). Meanwhile, we did not detect any off-target site, indicating a minor sgRNA-dependent off-target effect of BE4max *in vivo*.

**Figure 2.**
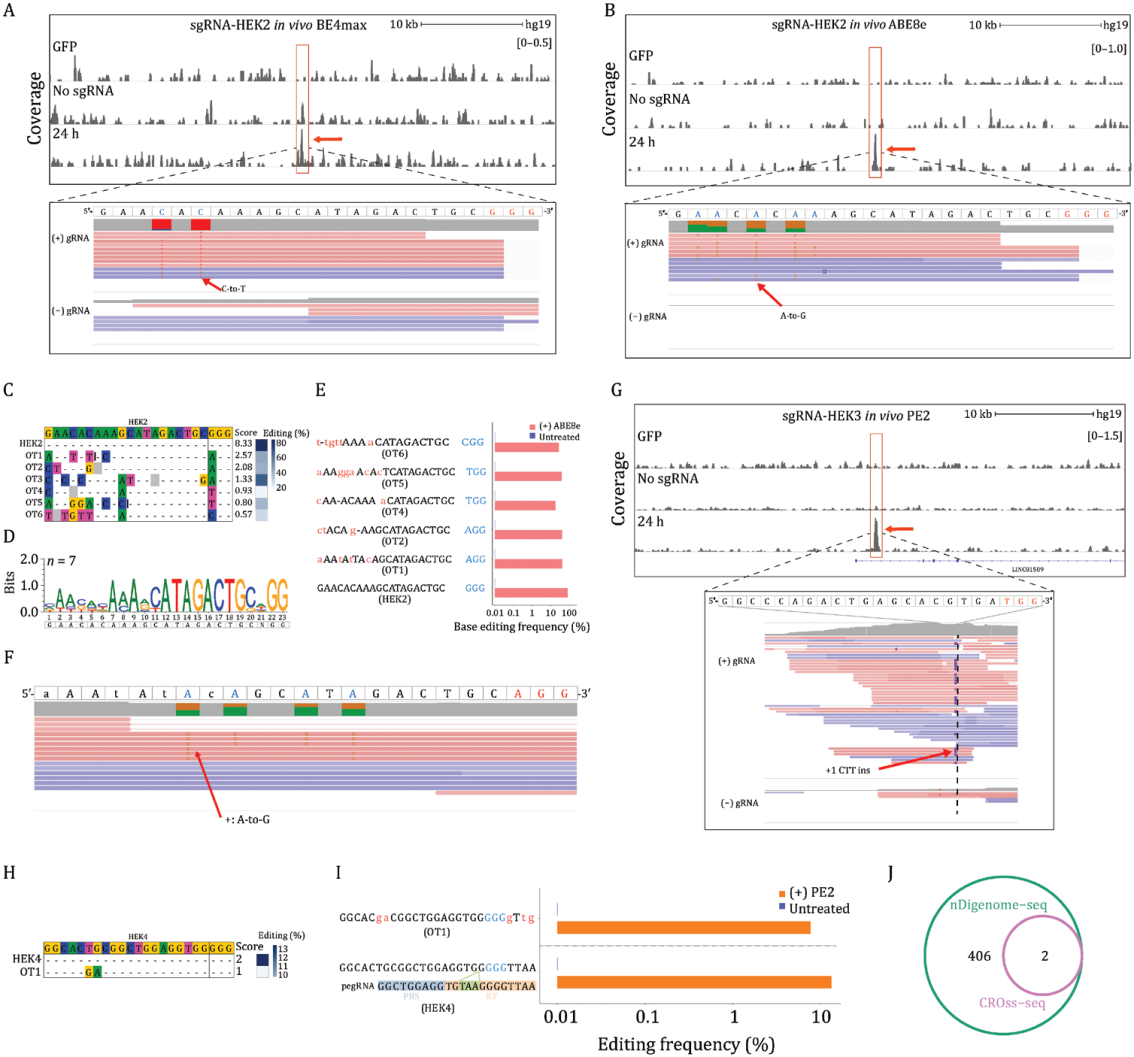
Genome-wide off-target sites induced by BE and PE revealed by CROss-seq. (A) The reads coverage from CROss-seq at the *HEK2* target locus of BE4max *in vivo*. Cells expressing GFP or the absence of sgRNA were used as controls. The *HEK2* on-target site is indicated by the red box. In the zoomed-in window, C-to-T mutations on the non-target strand are highlighted in color. The blue and red letters on the guide sequence represent the deaminated base and PAM sequence, respectively. (B) The reads coverage from CROss-seq at the *HEK2* target locus of ABE8e *in vivo*. Cells expressing GFP or the absence of sgRNA were used as controls. The *HEK2* on-target site is indicated by the red box. In the zoomed-in window, A-to-G mutations on the non-target strand are highlighted in color. The blue and red letters on the guide sequence represent the deaminated base and PAM sequence, respectively. (C) Off-target sequences and corresponding scores identified by ABE8e CROss-seq *in vivo*. The editing ratio for *HEK2* in HEK293T cells is shown on the right of each site. The on-target sequence is shown at the top of the alignment. Mismatched nucleotides are highlighted in color. Putative sgRNA bulges (gray) and target DNA bulges (black line) are shown. OT, off-target. (D) Sequence logos for *HEK2* sgRNA targeting site obtained using WebLogo by comparing DNA sequences at CROss-seq identified sites. (E) The base editing frequencies of off-target sites validated by ABE8e CROss-seq *in vivo*. Red and blue bars represent base editing frequencies obtained from cells expressing *HEK2* sgRNA or without sgRNA, respectively. PAM sequences are shown in blue. Mismatched bases are shown in red. OT, off-target. (F) The reads coverage from CROss-seq at the *HEK2* off-target OT1 of ABE8e *in vivo*. A-to-G mutations on the non-target strand are highlighted. The blue and red letters on the OT1 sequence represent the deaminated base and PAM sequence, respectively. (G) The reads coverage from CROss-seq at the *HEK3* target locus of PE2 *in vivo*. Cells expressing GFP or the absence of pegRNA were used as controls. The *HEK3* on-target site is indicated by the red box. The zoomed-in window shows that most reads contain a +1 CTT insertion (purple). The first nucleotide following the pegRNA-induced nick was counted as position +1. (H) Off-target sequences and corresponding scores identified by PE2 CROss-seq *in vivo*. The editing ratio for *HEK4* in HEK293T cells is shown on the right of each site. The on-target sequence is shown at the top of the alignment. Mismatched nucleotides are highlighted in color. OT, off-target. (I) The prime editing frequencies of off-target sites. Orange and blue bars represent prime editing frequencies obtained from cells expressing *HEK4* pegRNA or without pegRNA, respectively. PAM sequences are shown in blue. Mismatched bases are shown in red. OT, off-target. (J) A Venn diagram showing the number of off-target sites captured by CROss-seq and nDigenome-seq targeting the same *HEK4* locus in PE2 system.

Next, we applied CROss-seq to examine the off-target effects of the adenine base editor (ABE) (Gaudelli et al., 2017; Richter et al., 2020). Similar to CBE, the *in vitro* assay captured abundant reads distributed near the *HEK2* target site, showing a truncation signature resulting from nCas9 (Fig. S7E). We identified 25 off-target sites according to the CROSS score (Fig. S7F and S7G; Table S7). CROss-seq reads from the *in vivo* assay were also enriched near the target site, most of which contained an A-to-G editing signature (Fig. 2B). Meanwhile, six off-target sites were identified by the scoring program (Fig. 2C; Table S8). Consensus sequence analysis of the captured off-target sites agreed with the expected target site, and the PAM-proximal region was aligned with more accordance (Fig. 2D). Among these six off-target sites, five of them displayed A-to-G transition signal (Fig 2E and 2F; Fig. S9), indicating high reliability of CROss-seq in base editor systems.

Prime editor (PE) holds great promise in genome editing for its high specificity and expanded editing scope (Anzalone et al., 2019). Although nDigenome-seq (Kim et al., 2020) has been developed to assess the off-target effects of PE in a cell-free assay, the off-target effects of PE have not been tested in mammalian cells. To assess the off-target effects of PE *in vivo*, we transfected HEK293T cells with plasmids encoding PE2 and pegRNA targeting *HEK3*, and performed CROss-seq after 24 h. CROss-seq sequencing reads were evidently enriched near the target site, most of which contained a CTT insertion signature at the +1 position adjacent to the pegRNA-induced nick (Figs. 2G and S10A). Strikingly, only the on-target site could pass the CROSS-scoring cutoff (Figs. S10B, S10C and S11; Table S9). A recent study reported evident off-target effects of PE2 editing system by using nDigenome-seq (Kim et al., 2020). We therefore applied CROss-seq to investigate the editing with the pegRNA targeting the same locus *HEK4.* We identified the designed on-target site and one off-target site across the genome (Fig. 2H–J; Table S10). Of note, recent reports also indicated that PEs rarely induce off-target (Jin et al., 2021). These results together demonstrated the minimal off-target effects of PE.

In summary, we developed CROss-seq, a versatile, efficient, and cost-effective method for genome-wide off-target profiling in multiple genome editing systems. We applied CROss-seq both *in vivo* and *in vitro* to evaluate the off-target effects of Cas9 nuclease, base editors, and prime editor, while this method should also be adapted to other currently available or even potentially future CRISPR-mediated genome editors. Our results showed that the *in vivo* assay was more specific while *in vitro* assay was more sensitive, highlighting the necessity of coupling *in vivo* and *in vitro* assays to achieve a comprehensive and precise assessment of the off-target effects. CROss-seq also has its limitations. For example, the guanine density may influence the N_3_-kethoxal labeling efficiency, although the vast majority of human or mouse genome contains at least one G in the putative target regions (Fig. S12). To reduce the background noise, sample treated without sgRNA can be used as a negative control, which means CROss-seq can only identify gRNA-dependent editing events. We also anticipate applying CROss-seq in tissues and clinical samples with specific optimization. In conclusion, CROss-seq contributes to a deeper understanding of CRISPR-mediated tools in broader application scenarios.

## Supplementary Material

pwac018_suppl_Supplementary_Material

## Footnotes

This work was supported by the Ministry of Science and Technology of China to G.Z.L. (National Science and Technology Major Project, grant nos. 2018YFA0109100, 2019YFA0802203), National Natural Science Foundation of China to G.Z.L. (Grant Nos. 31922015, 31870808, 91753129), Natural Science Foundation of Guangdong Province to G.Z.L. (Grant No. 2018B030306044), and Guangdong Special Support Program to P.L. (2019BT02Y276).

We thank Dr Miao Yu and Dr Ye Fu for their insightful discussions about this work. We thank Dr Diana West-Szymanski for her help of synthesizing N_3_-kethoxal.

Tong Wu, Chuan He, and Guan-Zheng Luo; Methodology: Yan Li, Shenyao Zhi, Tong Wu, and Hong-Xuan Chen; Formal analysis and investigation: Yan Li, Shenyao Zhi, Tong Wu, Hong-Xuan Chen, Rui Kang and Dong-Zhao Ma; Writing: Yan Li, Shenyao Zhi, Tong Wu, Puping Liang, and Guan-Zheng Luo; Supervision: Zhou Songyang, Chuan He, Puping Liang, and Guan-Zheng Luo.

Sequencing data were deposited in NCBI GEO under accession number GSE182568.
