# Supplementary material for: Systematic identification of CRISPR off-target effects by CROss-seq
Source: Protein Cell. 2022 Jul 15;14(4):299–303. doi: 10.1093/procel/pwac018 (PMC10120991; doi:10.1093/procel/pwac018)
Supplement: pwac018_suppl_Supplementary_Material [file pwac018_suppl_supplementary_material.docx]

**Supplementary materials**

**Methods**

**Cell culture and transfection**

HEK293T cells were purchased from ATCC, which were cultured in Dulbecco's modified eagle medium (DMEM, Thermo) supplemented 10% fetal bovine serum (FBS, HyClone) and 1% penicillin/streptomycin (P/S, Invitrogen). Plasmids were transfected to HEK293T cells at about 75% confluency using PEI (Sigma-Aldrich, 408727).

**Plasmid construction**

px330 (#42230), ABE8e (#138489), pCMV-BE4max (#112093), and pET42b-BE3 (#87437) were purchased from Addgene. pET42b-ABE7.10, and pET28a-His-Cas9 were synthesized by Guangzhou IGE biotechnology Ltd. sgRNAs and pegRNAs were ligated to pUC19-Cas9 sgRNA expression vector. pegRNAs and sgRNAs sequences designed for different targets are listed in (Table S11).

**Protein purification and** **activity assessment**

pET42b-ABE7.10, pET42b-BE3, or pET28a-His-SpCas9 was expressed and purified as previously described (Liang et al.,2019). For SpCas9, ABE7.10, and BE3 activity assessment, we first purified PCR products (200 ng) containing the target sites. Then, the PCR products were incubated with 300 nM recombinant SpCas9, 900 nM sgRNA, and 4 μL 5 × CRB (CROss-seq reaction buffer) (50 mM MgCl_2_, 750 mM KCl, 100 mM HEPES (pH=7.4), 500 mM Sodium cacodylate) in a 20 μL reaction assay at 37 °C for 2 hours. ABE7.10 treated reaction mixture was purified using the PCR Cleanup Kit (Qiagen, 28506) before incubation with EndoV nuclease (ThermoFisher, EN0141) (1 U per 100 ng of PCR products) at 65 °C for 30 minutes. Same for the BE3 treated reaction mixture, except for incubating with USER enzyme (NEB, M5505S) (1 U per 100 ng of PCR products) at 37 °C for 1 hour. The digested products were resolved on a 2.5% agarose gel.

***In vitro* CROss-seq**

8 μg genomic DNA was extracted by using the DNeasy Blood & Tissue Kit (Qiagen, 69504) from HEK293T cells. Then, the gDNA was incubated with 300 nM recombinant proteins (SpCas9, ABE7.10, or BE3), 900 nM sgRNA, 0.05 mM N_3_-kethoxal (AccuraDX, ADX-NK-Soln), and 80 μL 5 × CRB buffer (50 mM MgCl2, 750 mM KCl, 100 mM HEPES (pH=7.4), 500 mM sodium cacodylate) in a 400 μL reaction mix for 2 hours at 37 °C. Treated genomic DNA was then purified using the DNeasy Blood & Tissue Kit (Qiagen, 69504) and eluted with 50 μL 25 mM K_3_BO_3_ (pH 7.0). The purified DNA (2 μg) was subjected to biotin click reaction, enrichment of N_3_-kethoxal-modified DNA, and library preparation subsequently.

***In vivo* CROss-seq**

6-well-plate HEK293T cells (7 × 10^5^/well) were transfected with px330 (#42230), ABE8e (#138489), pCMV-BE4max (#112093), and pUC19-Cas9 sgRNA expression plasmids (3 μg/well) using PEI (Sigma-Aldrich, 408727). To perform N_3_-kethoxal (AccuraDX, ADX-NK-Soln) labeling, 24 hours after transfection, the previous medium was removed and pre-warmed (37 °C) medium with 5 mM N_3_-kethoxal was added to incubate cells for 45 min at 37 °C. Genomic DNA was then extracted using the FastPure Cell/Tissue DNA Isolation MiniKit (Vazyme, DC102) and eluted with 100 μL 25 mM K_3_BO_3_ solution (pH 7.0). The extracted DNA (2 μg) was subjected to biotin click reaction, enrichment of N_3_-kethoxal-modified DNA, and library preparation subsequently.

**Click reaction, enrichment of N_3_-kethoxal-modified DNA, library preparation and sequencing**

2 µg genomic DNA was diluted in 85 µL of 25 mM K_3_BO_3_ (pH 7.0), combining with 10 µL 10 mM DBCO-PEG4-biotin (DMSO solution, Sigma, 760749), and 5 µL 20 × PBS (Sangon Biotech, B548117). Then, the mixture was incubated at 37 °C for 90 minutes with shaking at 500 rpm. Next, 5 µL RNase A (Takara, 2158) was added to the reaction mixture, followed by incubation at 37 °C for 15 minutes, shaking at 500 rpm. Biotinylated genomic DNA was purified from the reaction mixture using the DNA Clean & Concentrator-5 kit (Zymo, D4014), and eluted with 50 μL 25 mM K_3_BO_3_ (pH 7.0). 1 ug of the biotinylated product was adjusted to 50 µL using 25 mM K_3_BO_3_ (pH 7.0), and sheared on Diagenode Bioruptor Pico instrument to yield a fragment of 150-350 bp in size. 1 µL (~20 ng) of the fragmented DNA was used as input. Both input and the rest sample (referred to as IP) were repaired and ligated to adapters using the VAHTS Universal DNA Library Prep Kit for Illumina V3 (Vazyme, ND607) and purified with 0.6x DNA clean beads (Vazyme, N411). Then, the IP sample was subjected to biotin enrichment using Dynabeads MyOne Streptavidin C1 (Thermo, 65001). After 5 times washing, the product was released from beads via heating in 22.5 µL nuclease-free water at 95 °C for 10 minutes. Finally, the input and IP samples were amplified using the VAHTS HiFi Amplification Mix (Vazyme, N616). The purified libraries were sequenced on Illumina HiSeq X Ten platform to generate 10-20 million 150-bp paired-end reads.

**CROss-seq data processing and peak calling**

The quality of all reads was inspected using FastQC (v0.11.5, <http://www.bioinformatics.bbsrc.ac.uk/projects/fastqc>). Low-quality and adapter-containing reads were trimmed from raw data using Cutadapt (version 1.16) (Martin,2011). Clean reads were mapped to the reference human genome (hg19) using the bowtie2 aligner (version 2.3.4) with default settings (Langmead and Salzberg,2012). The SAM files were subsequently converted to the BAM file format, sorted, and indexed using SAMtools (version 1.6) (Li et al.,2009). Duplicate reads were removed using picard-tools (version 2.18.27, http://broadinstitute.github.io/picard/). BEDtools (v2.26.0) suite commands were used for format conversion (Quinlan and Hall,2010). These aligned reads were processed and converted into TDF (<https://software.broadinstitute.org/software/igv/TDF>) format, then visualized using the IGV Genome Browser (<https://software.broadinstitute.org/software/igv>) (Thorvaldsdóttir et al.,2013). Peaks were called using MACS (version 2.1.2) with default settings (-p 0.01) (Zhang et al.,2008). No-sgRNA (CRISPR alone) and GFP samples were used as a negative control. Common peaks between the edited and control sample were removed by BEDtools *intersect* command. Results were compared with ENCODE DAC Exclusion List Regions (Amemiya et al.,2019) (ENCODE #: ENCSR636HFF, i.e. blacklist) to filter out artifacts using BEDtools *intersect* command. The remaining peaks in the edited sample were potential off-target sites.

**Identification of off-target sites**

CROss-seq sequencing data was analyzed using a customized computational pipeline (Fig. S13). The scoring programs used to identify off-target cleavage sites or editing sites were illustrated in (Figs. S2, S8 and S11). Sequence reads with a mapping quality of 1 or greater were retained. To score putative editing off-target sites of BEs and PEs, the SNVs or INDELs were called using GATK (version 4.1.8.1) (McKenna et al.,2010). Candidate guide RNA off-target sites were obtained from Cas-OFFinder (v2.4) (Bae et al.,2014). For putative Cas9 cleavage sites, we first identified all loci with three or more reads starting (for reverse PAM-strand) or ending (for forward PAM-strand) at the same point, or having at most three bp overlap. For putative editing sites of BE and PE, we identified all loci with intended editing, and filtered the SNP/INDEL sites. Then we applied the scoring program to score each site, filtering out the sites in ENCODE blacklist and/or not in the filtered peaks, and checking the presence of PAM sequences and protospacer sequences. Finally, we ranked and selected the off-target sites according to the CROSS score. For Cas9 system, we obtained a ranking of each site, and kept those with scores >= 5 and cleavage ratio >= 0.3. For BEs and PEs system, we sorted the scores of each site, and selected sites with scores >= 0.5 and edited ratio >= 0.3. The amount of mismatch and bulge between putative guide sequence and protospacer were counted. Up to 6 mismatches and 1 bulge (DNA or RNA) were allowed to present in the final sequence. Multiple sequence alignments of sgRNA sequences and their off-targets were generated and visualized using JalView (version 2.11.1.4) (Waterhouse et al.,2009).

**Targeted deep sequencing and evaluation of the editing efficiency**

Genomic DNA was purified from HEK293T cells using the DNeasy Blood & Tissue Kit (Qiagen, 69504). The on-target and potential off-target sites were amplified with barcode-containing primers using the KOD PCR kit (Toyobo, KMM-101) to generate a deep sequencing library. The 150 bp paired-end libraries were then deep sequenced on NovaSeq 6000 (Illumina) platform. Primer information can be found in (Tables S12 and S13). The indel efficiencies of Cas9 were analyzed using CRISPResso2 (version 2.0.45) (Clement et al.,2019). Indels located within ten bp of the putative Cas9 cleavage site were counted as mutations induced by Cas9. The substitution efficiencies of BE were calculated using the CRISPResso2 base editor module.

**Chromatin accessibility analysis**

To determine whether off-target sites identified by both *in vitro* and *in vivo* CROss-seq were associated with chromatin accessibility, we analyzed DNase-seq data of HEK293T cells from ENCODE (ENCODE #: ENCSR000EJR). We selected *in vitro* and *in vivo* SpCas9 off-target sites of *HBG* sgRNA to analyze their differential enrichment in open chromatin regions. Nonoverlapping regions generated by applying a 1-kb sliding window on the human genome were defined as control regions. We randomly selected 38 or 59 regions as the control group for *in vivo* and *in vitro* off-target sites, respectively. To analyze the hypersensitivity levels of DNase I towards these sites, the reads count of DNase-seq for off-target sites as well as for the control regions were normalized to the length of the genomic window. Then the number of off-target sites overlapping with DNase I-hypersensitive sites was calculated using BEDtools *intersect* command. To analyze overlap between off-target sites and control genomic regions, 1-kb control regions were randomly generated 1,000 times, and the percentage for each computational experiment was calculated.


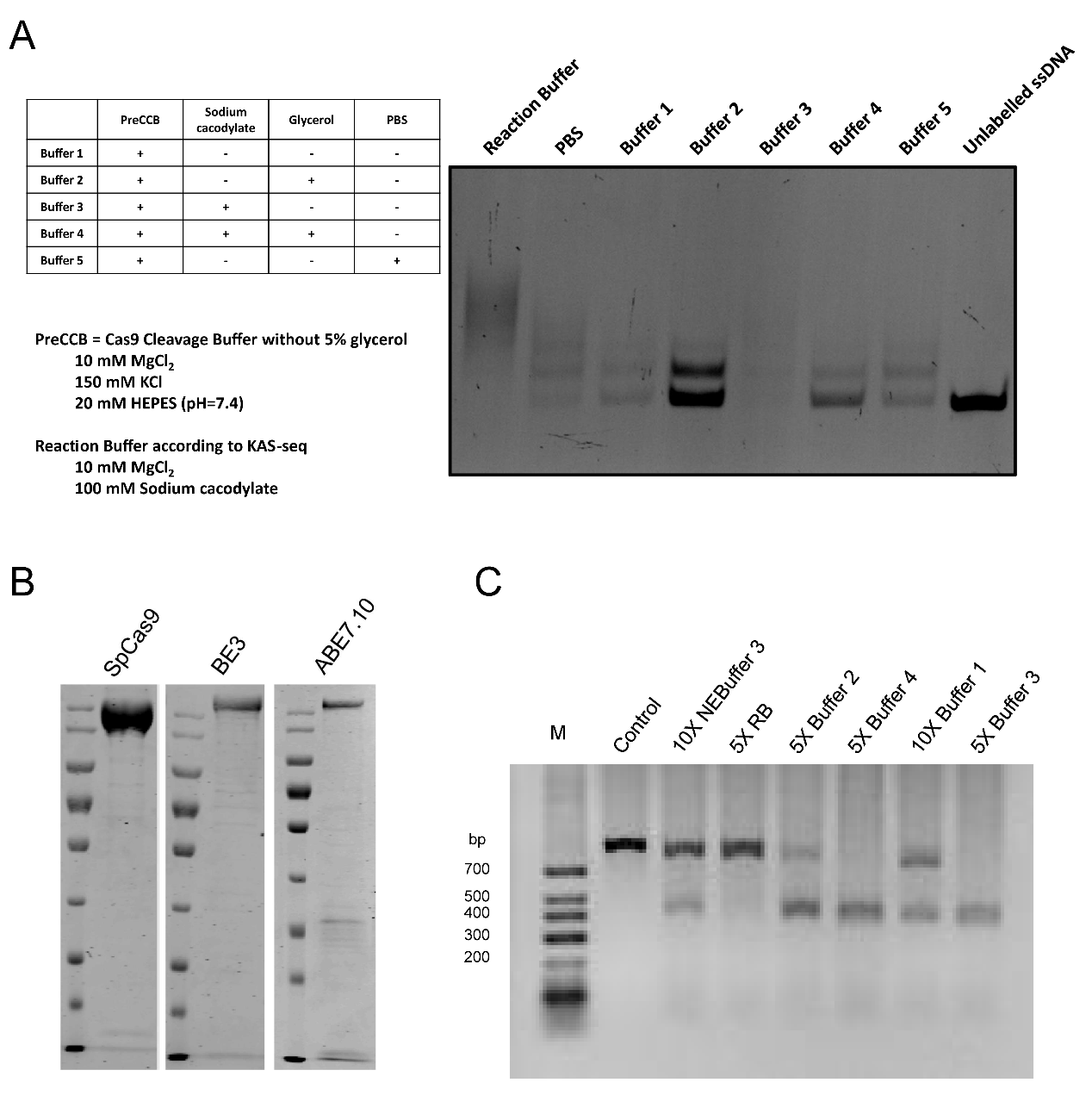


**Figure S1**. **Optimization of the reaction buffer for *in vitro* CROss-seq**. (A) Different reaction buffer displays different labeling efficiency of N_3_-kethoxal (the buffer is prepared based on the SITE-seq CCB buffer). Buffer 3 (PreCCB with Sodium cacodylate) shows the highest efficient labeling efficiency. (B) Recombinant Cas9, BE3, and ABE7.10 proteins were purified from bacteria and examined by SDS-PAGE and coomassie blue staining. (C) Buffer 3 did not influence the cleavage efficiency of Cas9.


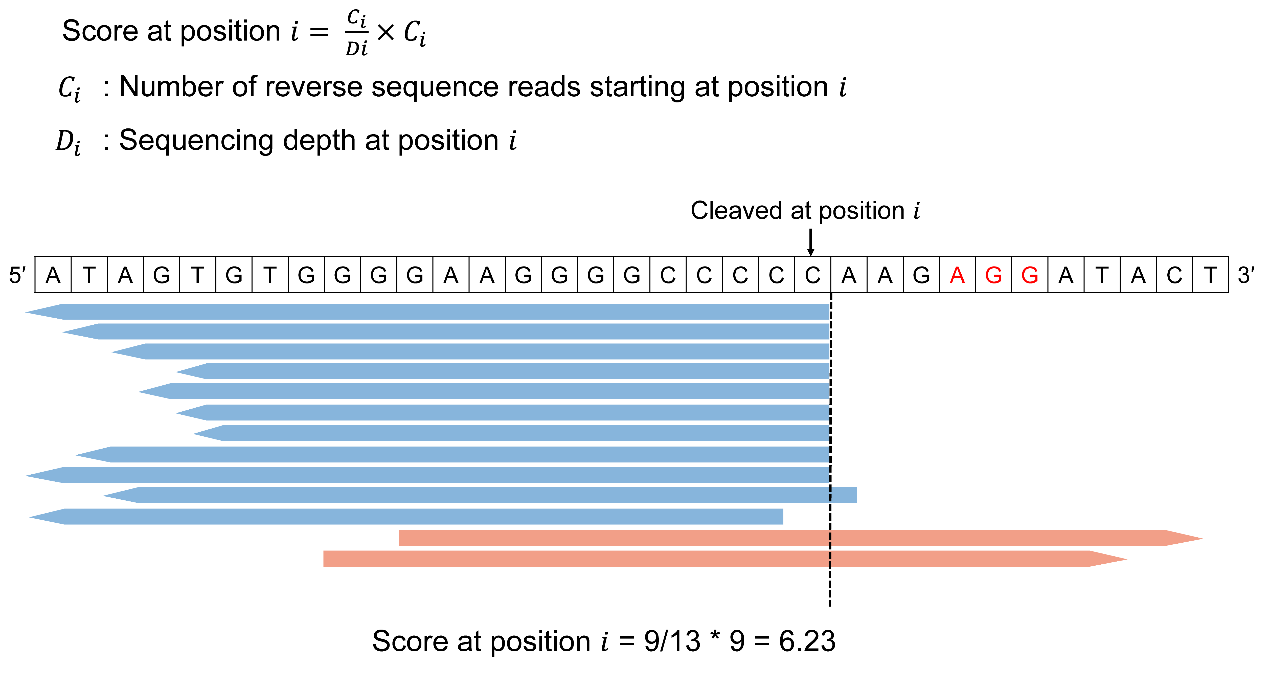


**Figure S2**. **DNA cleavage scoring system for CROss-seq analysis of Cas9, or BE *in vitro*.**


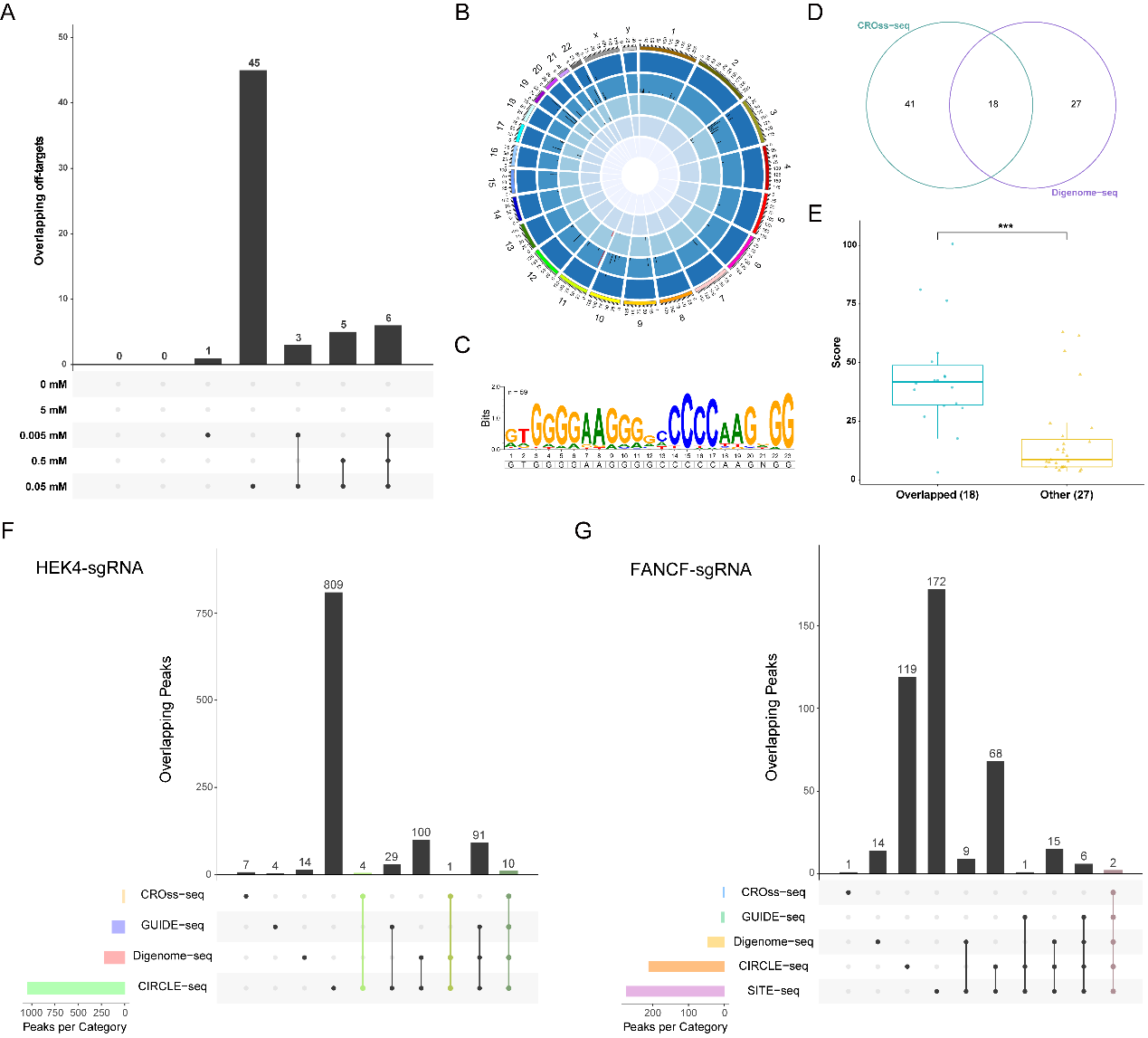


**Figure S3**. **Comparisons of CROss-seq with GUIDE-seq, Digenome-seq, CIRCLE-seq and SITE-seq.** (A) The number of off-target sites detected when different concentrations of N_3_-kethoxal was used. Cas9 was targeted to the *HBG* locus. (B) Genome-wide circos plots representing the SpCas9 CROSS score for *HBG* *in vitro.* Samples were treated with different working concentration of N_3_-kethoxal (from inside to outside, 0 mM (gray), and different N_3_-kethoxal concentrations (blue, 5mM, 0.5mM, 0.05mM, 0.005mM)). The red lines indicate the on-target sites. (C) Sequence logo for *HBG* sgRNA targeting site obtained using WebLogo by comparing DNA sequences at CROss-seq identified sites (0.05 mM N_3_-kethoxal). (D) A Venn diagram showing the number of *in vitro* cleavage sites captured by CROss-seq and Digenome-seq targeting the same *HBG* locus. (E) DNA cleavage score of Digenome-seq off-target sites, the scores of the 18 sites that overlapped with those from CROss-seq are significantly higher than others’. The statistical significance was calculated using a two-tailed unpaired t-test (* p < 0.05, ** p < 0.01, *** p < 0.001). (F) The comparisons of off-target sites detected by CROss-seq, GUIDE-seq, Digenome-seq and CIRCLE-seq. Cas9 was targeted to the same *HEK4* locus. (G) The comparisons of off-target sites detected by CROss-seq, GUIDE-seq, Digenome-seq, CIRCLE-seq and SITE-seq. Cas9 was targeted to the same *FANCF* locus.


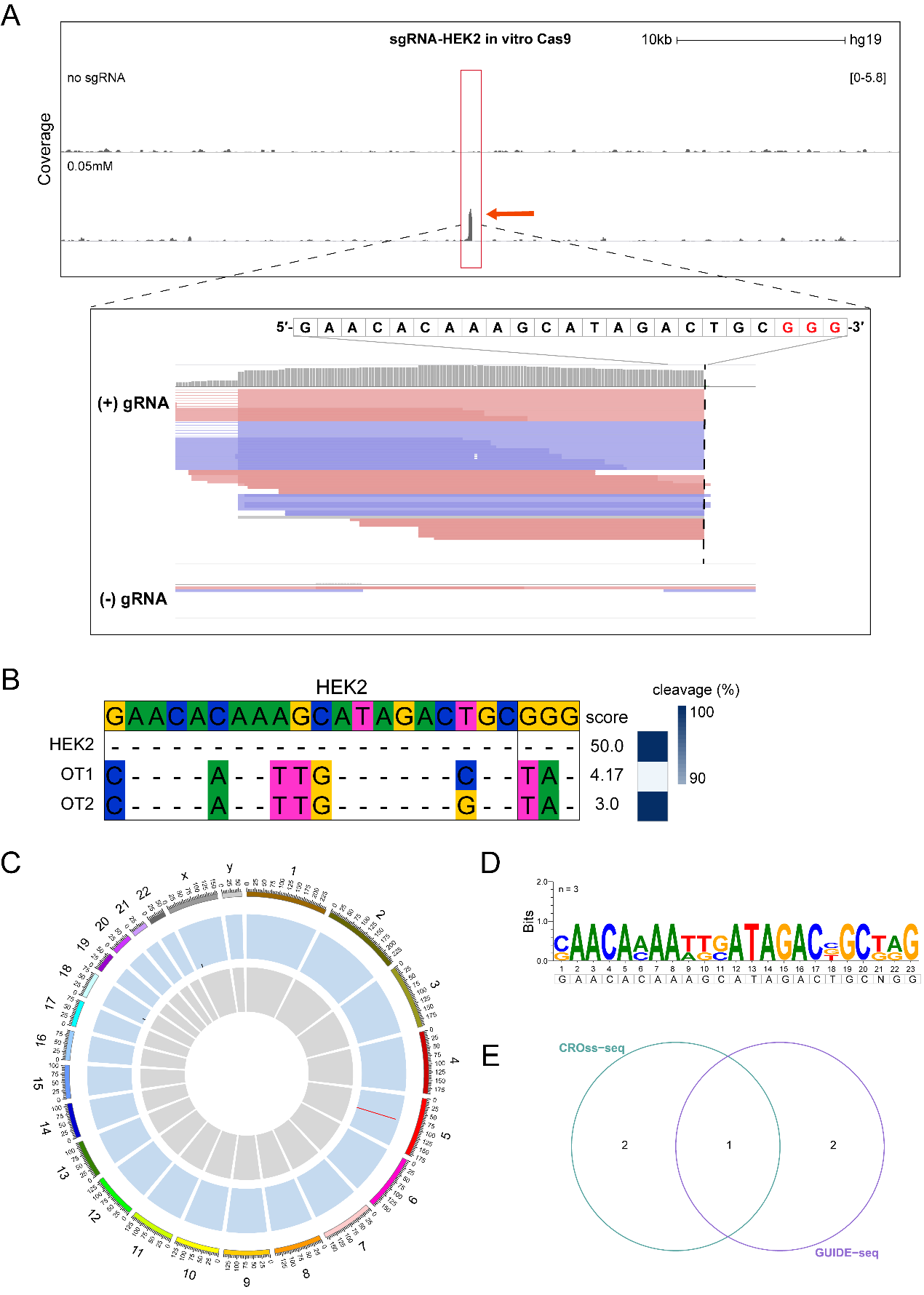


**Figure S4**. **Off-target sites induced by the *HEK2*-targeted SpCas9 identified by CROss-seq *in vitro*.** (A) The reads coverage from CROss-seq at the *HEK2* target locus of SpCas9 *in vitro*. The *HEK2* on-target site is indicated by the red box. The zoomed-in window shows that most reads end in the SpCas9 cleavage site. (B) Off-target sequences and corresponding scores identified by SpCas9 CROss-seq *in vitro*. The cleavage ratios for *HEK2* in HEK293T cells are shown on the right of each site. The on-target sequence is shown at the top of the alignment. Mismatched nucleotides are highlighted in color. Putative sgRNA bulges (gray) and target DNA bulges (black line) are shown. OT, off-target. (C) Genome-wide circos plots representing SpCas9 CROSS score for *HEK2* *in vitro* (from inside to outside, without sgRNA (gray), with sgRNA and treated with 0.05mM N_3_-kethoxal concentration (blue)). The red line indicated the on-target sites. (D) Sequence logos for *HEK2* sgRNA targeting site obtained using WebLogo by comparing DNA sequences at CROss-seq identified sites. (E) A Venn diagram showing the number of *in vitro* cleavage sites captured by CROss-seq and GUIDE-seq with the *HEK2*-targeted Cas9.


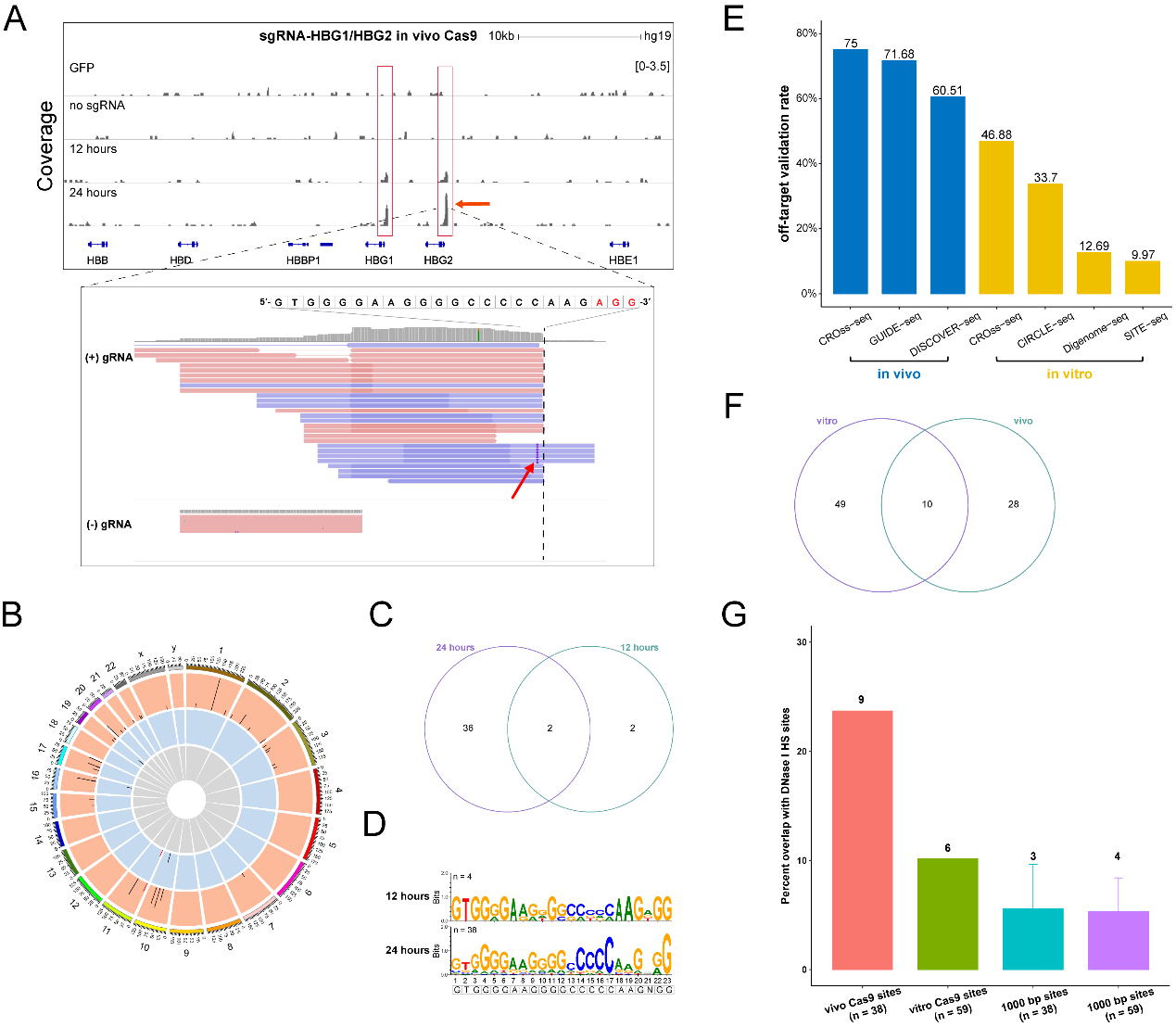


**Figure S5**. **Off-target effects introduced by SpCas9 *in vitro* and *in vivo* were evaluated by CROss-seq parallelly.** (A) The reads coverage from CROss-seq at the *HBG* target locus of cells transfected for different time (12 and 24 hours). Cells expressing GFP or the absence of sgRNA were used as controls. The *HBG1* and *HBG2* on-target sites are indicated by the red box. The zoomed-in window shows that most reads end in the SpCas9 cleavage site. An indel is indicated by the arrow. (B) Genome-wide circos plots representing SpCas9 CROSS score for *HBG* *in vivo*. Cells were transfected for different time (from inside to outside, without sgRNA (gray), 12 hours (blue), 24 hours (orange)). The red line indicates the on-target sites. (C) A Venn diagram showing the number of *in vivo* cleavage sites captured by CROss-seq with the *HBG*-targeted SpCas9. Samples were either transfected for 12 hours or 24 hours. (D) Sequence logos for *HBG* sgRNA targeting site obtained using WebLogo by comparing DNA sequences at CROss-seq identified sites (transfect 12 hours and 24 hours). (E) The off-target validation rate of *in vivo* methods and *in vitro* methods respectively. Comparisons of *in vivo* CROss-seq with GUIDE-seq and DISCOVER-seq. Comparisons of *in vitro* CROss-seq with Digenome-seq, CIRCLE-seq and SITE-seq. (F) A Venn diagram showing the number of *in vitro* and *in vivo* cleavage sites captured by CROss-seq with the *HBG*-targeted SpCas9. (G) The percentage and the number of overlap between SpCas9 off-target sites and ENCODE-defined DNAse I hypersensitivity (HS) sites *in vitro* and *in vivo*. Error bars indicate the s.d. of 1,000 computational simulations, randomly selected 1000-bp genomic regions.


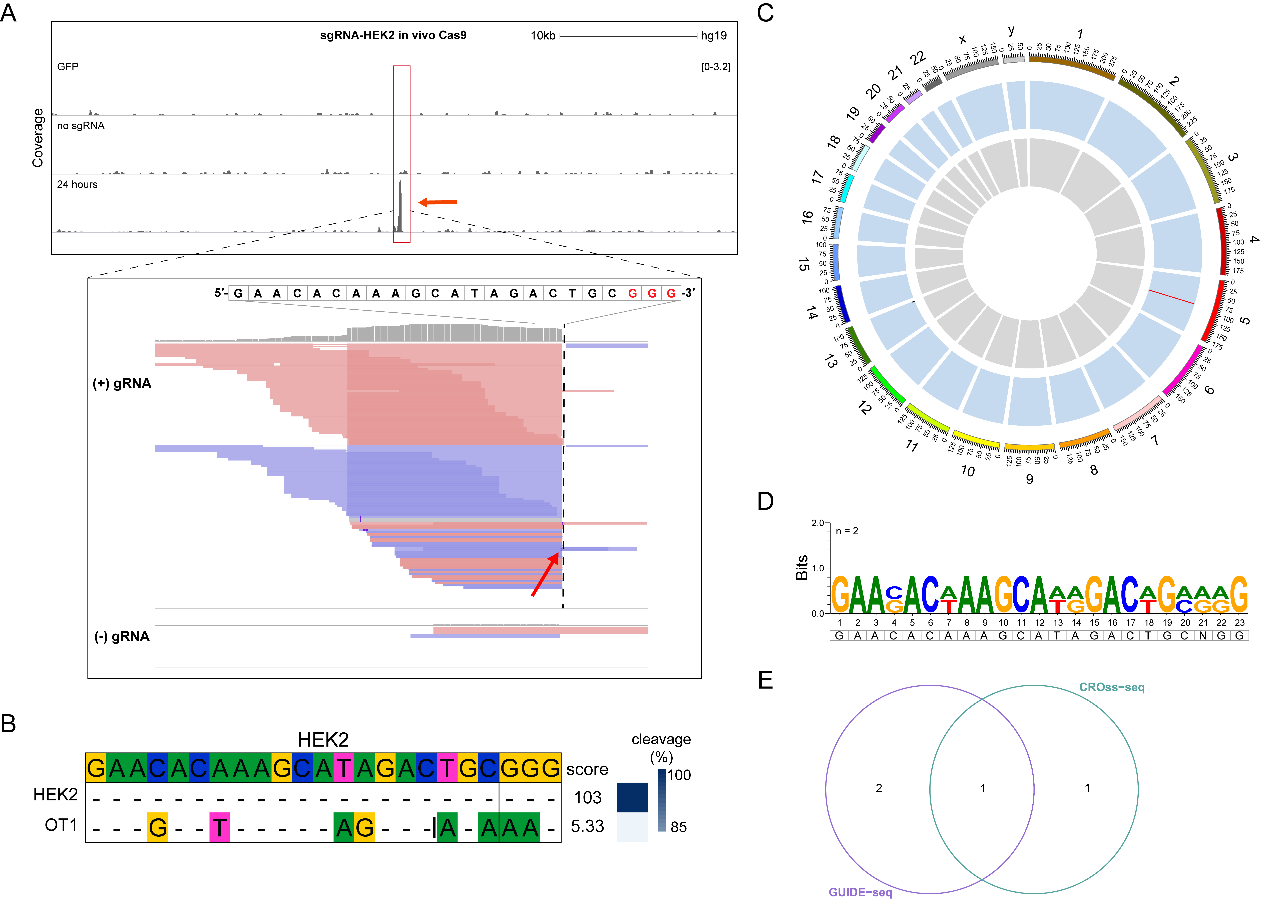


**Figure S6**. **Off-target sites induced by the *HEK2*-targeted SpCas9 identified by CROss-seq *in vivo*.** (A) The reads coverage from CROss-seq at the *HEK2* target locus of SpCas9 *in vivo*. The *HEK2* on-target site is indicated by the red box. The zoomed-in window shows that most reads end in the SpCas9 cleavage site. (B) Off-target sequences and corresponding scores identified by SpCas9 CROss-seq *in vivo*. The cleavage ratios for *HEK2* in HEK293T cells are shown on the right of each site. The on-target sequence is shown at the top of the alignment. Mismatched nucleotides are highlighted in color. Putative sgRNA bulges (gray) and target DNA bulges (black line) are shown. OT, off-target. (C) Genome-wide circos plots representing SpCas9 CROSS score for *HEK2* *in vivo* (from inside to outside, without sgRNA (gray), with sgRNA(blue)). The red line indicated the on-target sites. (D) Sequence logos for *HEK2* sgRNA targeting site obtained using WebLogo by comparing DNA sequences at CROss-seq identified sites. (E) A Venn diagram showing the number of *in vivo* cleavage sites captured by CROss-seq and GUIDE-seq with the *HEK2*-targeted SpCas9.


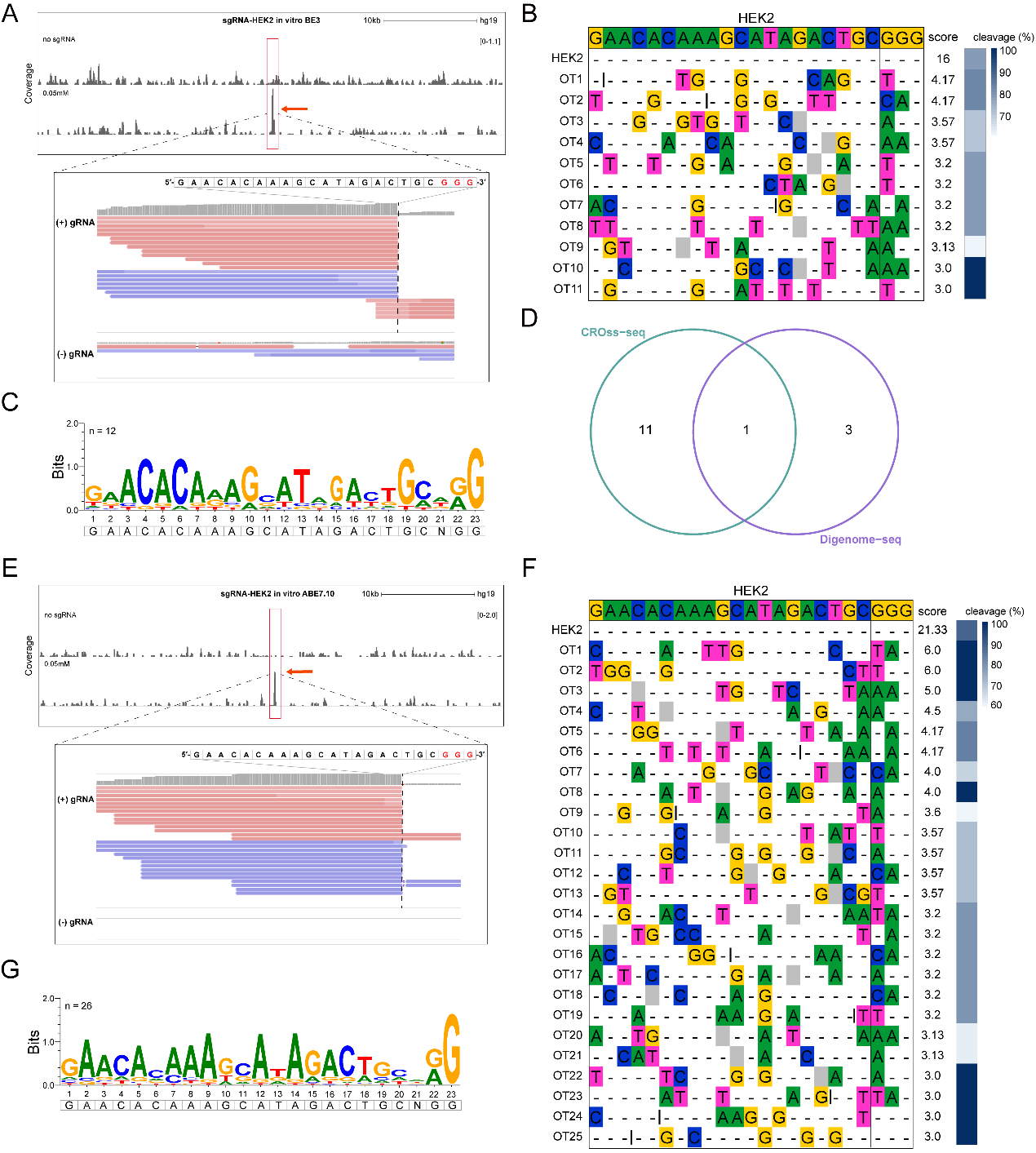


**Figure S7**. **Genome-wide off-target sites induced by BE revealed by *in vitro* CROss-seq.** (A) The reads coverage from CROss-seq at the *HEK2* target locus of BE3 *in vitro*. Samples without sgRNA supplement were served as controls. The *HEK2* on-target site is indicated by the red box. The zoomed-in window shows that most reads end in the nCas9 (D10A) cleavage site. (B) Off-target sequences and corresponding scores identified by BE3 CROss-seq *in vitro*. The cleavage ratios for *HEK2* in HEK293T cells are shown on the right of each site. The on-target sequence is shown at the top of the alignment. Mismatched nucleotides are highlighted in color. Putative sgRNA bulges (gray) and target DNA bulges (black line) are shown. OT, off-target. (C) Sequence logos for *HEK2* sgRNA targeting site obtained using WebLogo by comparing DNA sequences at CROss-seq identified sites. (D) A Venn diagram showing the number of *in vitro* cleavage sites captured by CROss-seq and Digenome-seq using the *HEK2*-targeted BE3. (E) The reads coverage from CROss-seq at the *HEK2* target locus of ABE7.10 *in vitro*. Samples without sgRNA supplement were served as controls. The *HEK2* on-target site is indicated by the red box. The zoomed-in window shows that most reads end in the nCas9 (D10A) cleavage site. (F) Off-target sequences and corresponding scores identified by ABE7.10 CROss-seq *in vitro*. The cleavage ratios for *HEK2* in HEK293T cells are shown on the right of each site. The on-target sequence is shown at the top of the alignment. Mismatched nucleotides are highlighted in color. Putative sgRNA bulges (gray) and target DNA bulges (black line) are shown. OT, off-target. (G) Sequence logos for *HEK2* sgRNA targeting site obtained using WebLogo by comparing DNA sequences at CROss-seq identified sites.


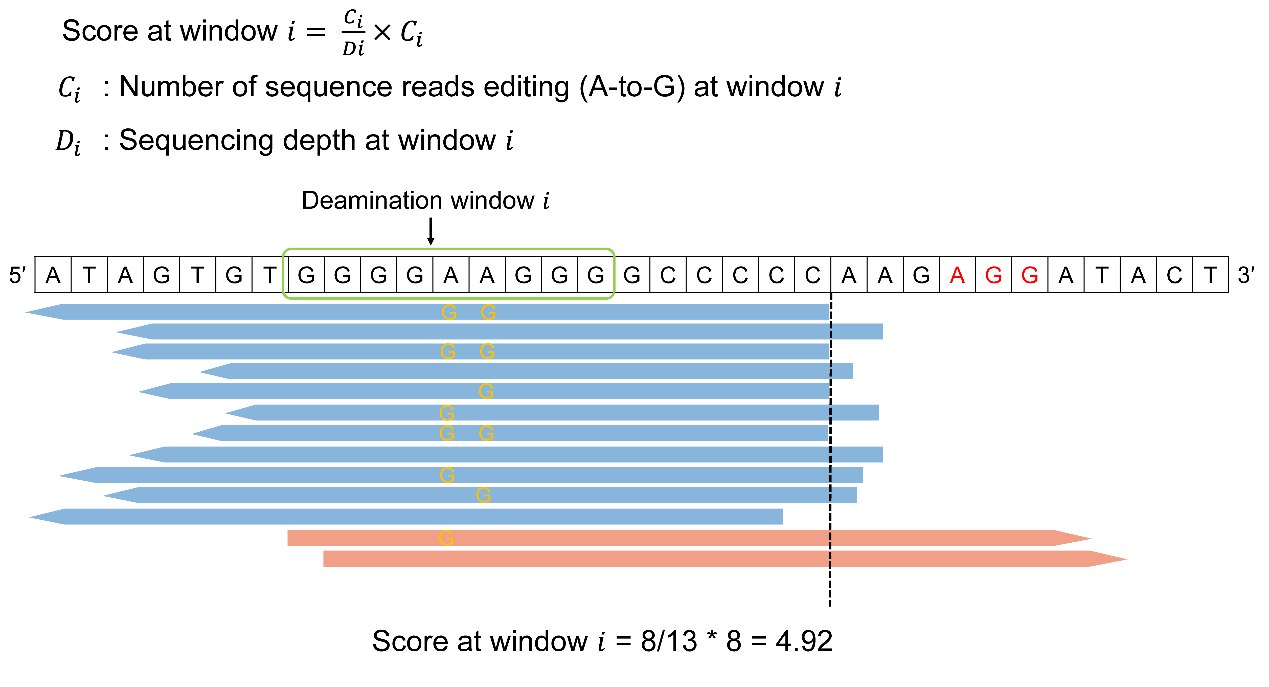


**Figure S8**. **DNA editing scoring system for CROss-seq analysis of BE *in vivo*.**


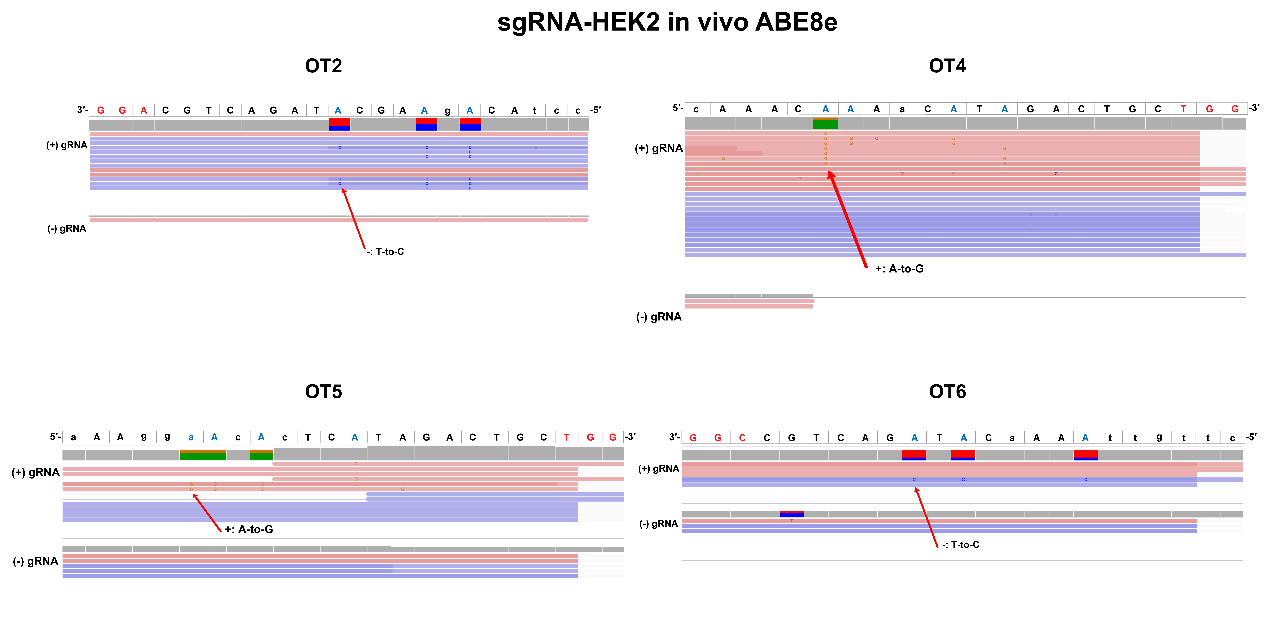


**Figure S9**. **Representative off-target edits on the non-target strand induced by *HEK2*-targeted ABE8e (or PAM strand).** The on-target sequence is shown at the top of the window. Mismatched nucleotides are shown in lowercase. Positions 21–23 are recognized as the PAM region and are highlighted in red (for forward non-target strand, or positions 1-3 are recognized as the PAM region for reverse non-target strand). The edited bases within the deamination window are highlighted in blue.


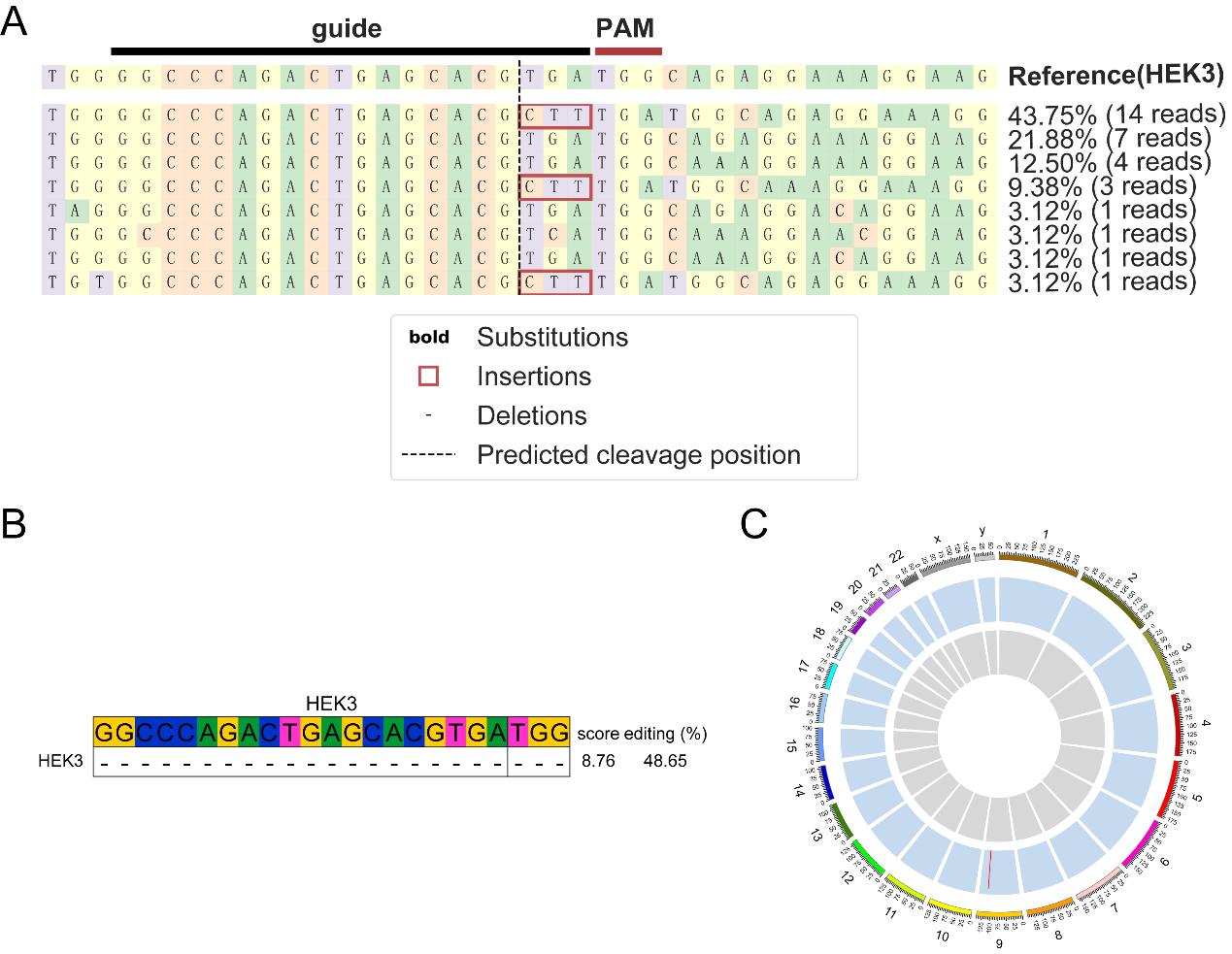


**Figure S10**. **Off-target sites induced by PE revealed by CROss-seq.** (A) Example *HEK3* site allele tables from genomic DNA samples isolated from HEK293T cells after editing with PE2. The reference *HEK3* sequence from this region is at the top. Allele tables are shown for a +1 CTT insertion at *HEK3* using PE2. Allele frequencies and corresponding Illumina sequencing read counts are shown for each allele. (B) Off-target sequences and corresponding scores identified by PE2 CROss-seq *in vivo*. The editing ratios for *HEK3* in HEK293T cells are shown on the right of each site. The on-target sequence is shown at the top of the alignment. Mismatched nucleotides are highlighted in color. Putative sgRNA bulges (gray) and target DNA bulges (black line) are shown. OT, off-target. (C) Genome-wide circos plots representing PE2 CROSS score for *HEK3 in vivo* (from inside to outside, without sgRNA (gray), transfected with *HEK3* sgRNA (blue)). The red line indicates the on-target sites.


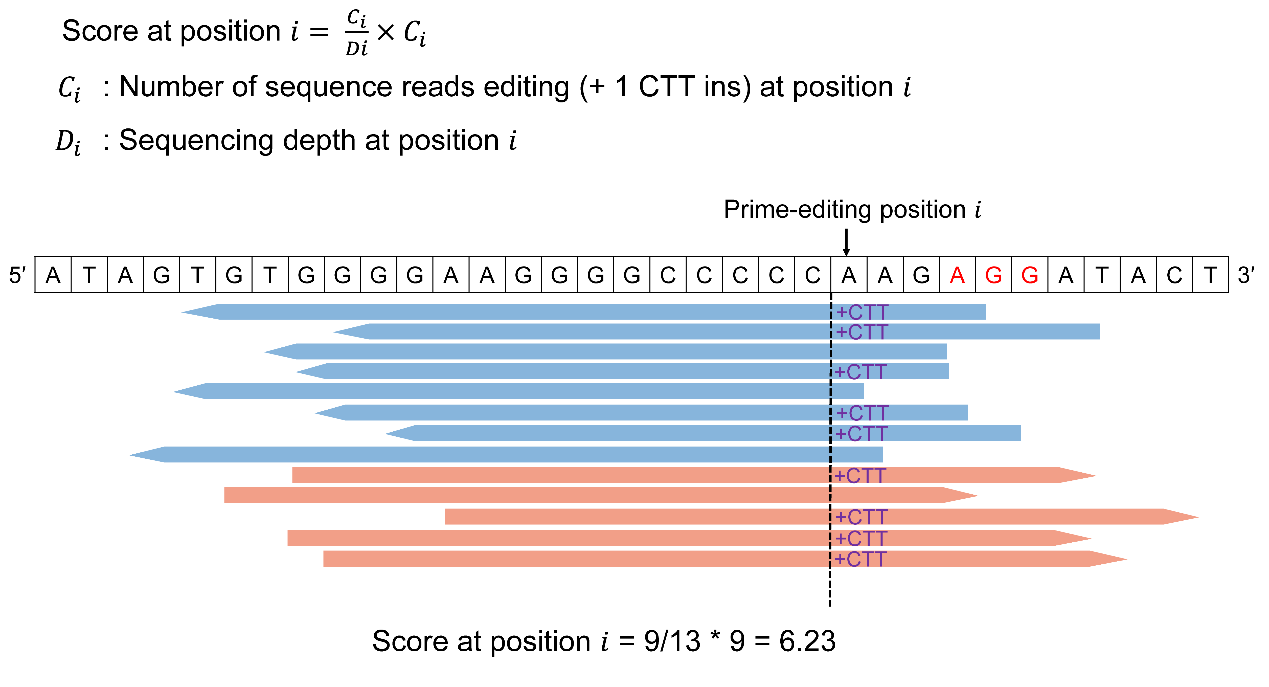


**Figure S11**. **DNA editing scoring system for CROss-seq analysis of PE *in vivo*.**


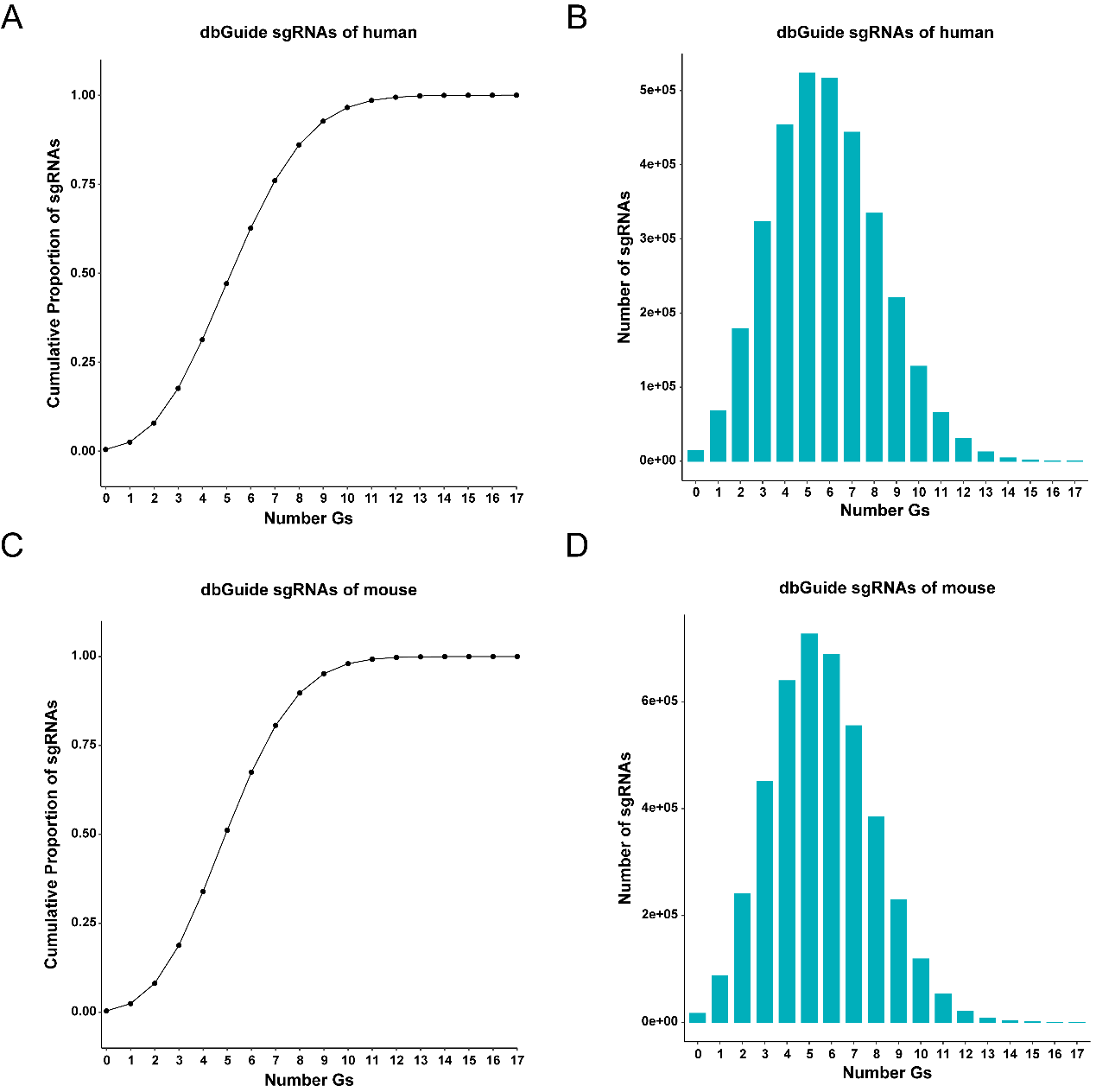


**Figure S12**. **Evaluation of the frequency of G nucleotides within sgRNA under the human or mouse genomic background.** (A) Cumulative fraction of G nucleotides number of sgRNAs under the human genomic background (hg19). (B) Absolute number of G nucleotides of sgRNAs under the human genomic background (hg19). (C) Cumulative fraction of G nucleotides number of sgRNAs under the mouse genomic background (mm10). (D) Absolute number of G nucleotides of sgRNAs under the mouse genomic background (mm10).


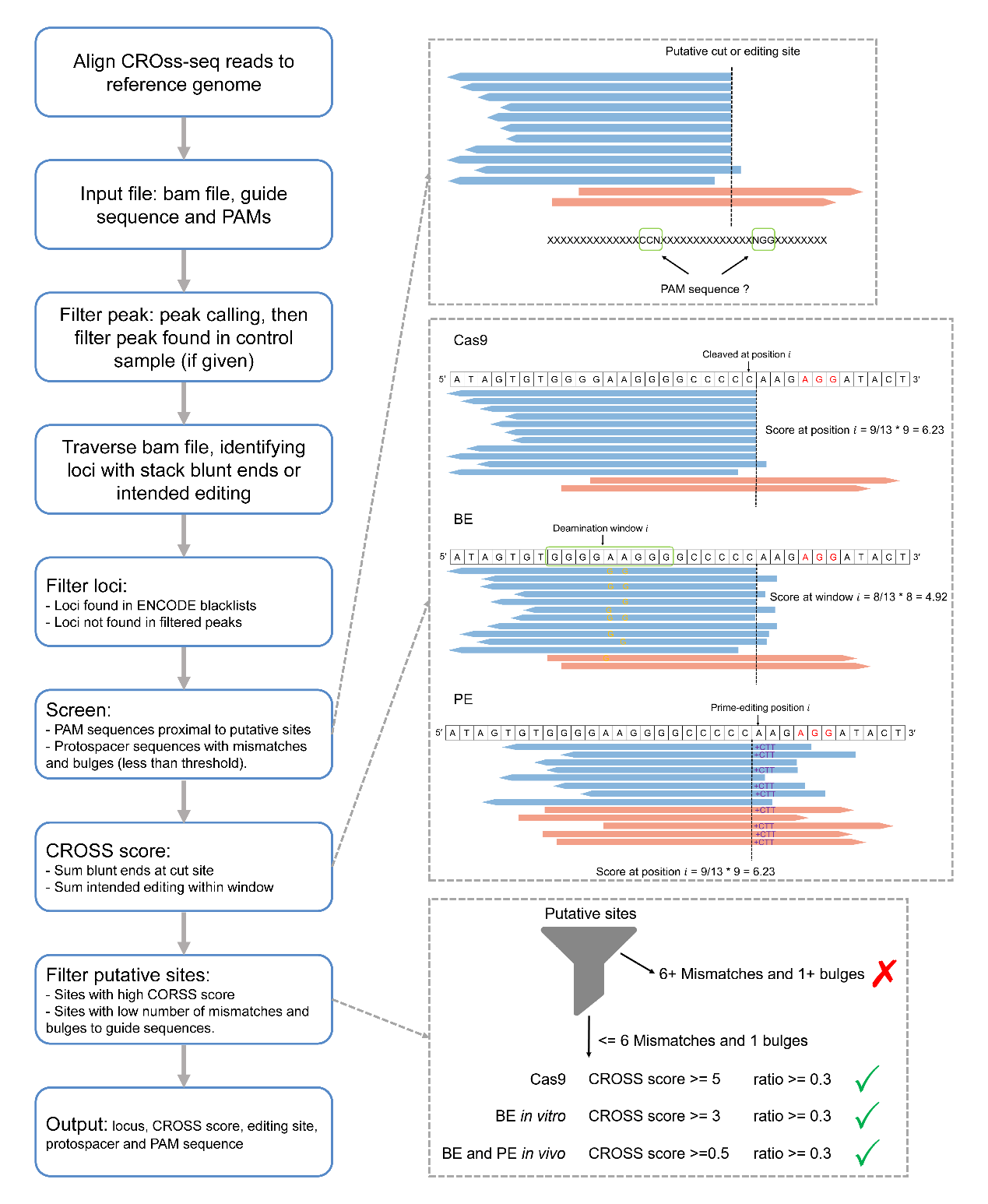


**Figure S13. CROss-seq analysis workflow.** Workflow of the CROss-seq computational pipeline (see **Methods** for details).

**Table S1.** **CROss-seq captured sites using *HBG* gRNA and Cas9 *in vitro*.**

| **N_3_-kethoxal** | **Site name** | **Peak Position** | | | **off-target site** | | | | **DNA sequence at off-target site** | **CROSS score** | **Bulge** | **Validated** |
| --- | --- | --- | --- | --- | --- | --- | --- | --- | --- | --- | --- | --- |
|  |  | **chr** | **start** | **end** | **chr** | **start** | **end** | **strand** |  |  |  |  |
| 0.05mM | OT1 | chr19 | 53377889 | 53378162 | chr19 | 53377883 | 53377905 | - | GTGGGaAAaaGt-CCCCAAGTGG | 82 | RNA | No |
|  | HBG1 | chr11 | 5271004 | 5271295 | chr11 | 5271278 | 5271301 | + | GTGGGGAAGGGGCCCCCAAGAGG | 73.05194805 | X | Yes |
|  | OT2 | chr22 | 43265282 | 43265596 | chr22 | 43265579 | 43265602 | + | GgGGaGAAGGGaaCCCCAAGGGG | 65.05797101 | X | No |
|  | OT3 | chr3 | 13705837 | 13706121 | chr3 | 13705831 | 13705853 | - | GT-GGGAtGGGGtCCCCAAGTGG | 63.22535211 | RNA | Yes |
|  | HBG2 | chr11 | 5275922 | 5276219 | chr11 | 5276202 | 5276225 | + | GTGGGGAAGGGGCCCCCAAGAGG | 57.09459459 | X | Yes |
|  | OT4 | chr19 | 33976056 | 33976345 | chr19 | 33976056 | 33976078 | - | GaGGGG-AGGGaCCCtCAAGAGG | 56.52941176 | RNA | No |
|  | OT5 | chr3 | 4746490 | 4746727 | chr3 | 4746484 | 4746507 | - | aTGaGGAAGcGaCCCCCAAGAGG | 48 | X | Yes |
|  | OT6 | chr10 | 96955747 | 96956017 | chr10 | 96955741 | 96955763 | - | GTGGGG-AGGGcaCCCCAAGAGG | 46.17307692 | RNA | No |
|  | OT7 | chr12 | 48137292 | 48137556 | chr12 | 48137538 | 48137562 | + | aTGGTGGgAGGGatCCCCAAGAGG | 46 | DNA | No |
|  | OT8 | chr3 | 34824511 | 34824806 | chr3 | 34824505 | 34824527 | - | GT-aGGAAGGGGCtCCCAAGAGG | 43 | RNA | Yes |
|  | OT9 | chr21 | 47118130 | 47118432 | chr21 | 47118124 | 47118146 | - | aaGGGGAAGGaG-CCCCAAGAGG | 40 | RNA | No |
|  | OT10 | chr17 | 27910706 | 27910999 | chr17 | 27910982 | 27911005 | + | GgGaGGAAGGGatCCCCAAGGGG | 39 | X | No |
|  | OT11 | chrX | 12298825 | 12299093 | chrX | 12298819 | 12298841 | - | GT-GGGAAaGGaCCCCCAAtGAG | 38 | RNA | No |
|  | OT12 | chr9 | 21121874 | 21122162 | chr9 | 21122146 | 21122168 | + | GT-GaGAAGGaGCCCaCAAGTGG | 35.02702703 | RNA | No |
|  | OT13 | chr3 | 48687123 | 48687395 | chr3 | 48687379 | 48687401 | + | GTGGGG-AGGGGCCCCCAAGCAG | 33.10810811 | RNA | No |
|  | OT14 | chr20 | 24950448 | 24950680 | chr20 | 24950443 | 24950465 | - | GatGGGgAaGGGCCCCC-AGGGG | 31.24324324 | RNA | No |
|  | OT15 | chr15 | 84049326 | 84049568 | chr15 | 84049552 | 84049574 | + | GTGGGG-AGGcGCCCtCAAGTGG | 31.03030303 | RNA | No |
|  | OT16 | chr7 | 27274996 | 27275270 | chr7 | 27275253 | 27275276 | + | GatGGaAAGGGaCCCtCAAGGGG | 31 | X | No |
|  | OT17 | chr10 | 73282209 | 73282453 | chr10 | 73282203 | 73282225 | - | GTGGGG-AGtGGCCCCCAAGAGG | 30 | RNA | Yes |
|  | OT18 | chr1 | 2.03E+08 | 2.03E+08 | chr1 | 202936273 | 202936295 | + | aTGGGG-AGGGGtCCCCcAGTGG | 25 | RNA | No |
|  | OT19 | chr8 | 1.29E+08 | 1.29E+08 | chr8 | 129326154 | 129326176 | - | GTGGGaAAGGGG-CCCCAtGGGG | 23.14814815 | RNA | No |
|  | OT20 | chr3 | 23491399 | 23491589 | chr3 | 23491393 | 23491416 | - | taGGGGgAGGGGtCCCCAtGTGG | 22 | X | Yes |
|  | OT21 | chr22 | 18919314 | 18919595 | chr22 | 18919578 | 18919601 | + | GTGGtaAAGGaaCCCCaAAGGGG | 21.16 | X | No |
|  | OT22 | chr11 | 1.2E+08 | 1.2E+08 | chr11 | 119694416 | 119694440 | + | GTGGTGaAAGGGtCCCaCAAGAGG | 20.57142857 | DNA | No |
|  | OT23 | chr3 | 1.23E+08 | 1.23E+08 | chr3 | 123001313 | 123001336 | + | aTGGGagAGGGGtCCCCAtGGGG | 20 | X | Yes |
|  | OT24 | chr17 | 79276568 | 79276822 | chr17 | 79276562 | 79276585 | - | acGGGGAAGaGaCCCCCAgGAGG | 18.18181818 | X | No |
|  | OT25 | chr1 | 13794531 | 13794733 | chr1 | 13794716 | 13794739 | + | tTGaGGAAGGGGaCCCCAAGAGG | 18.18181818 | X | No |
|  | OT26 | chr17 | 80533914 | 80534191 | chr17 | 80534174 | 80534197 | + | tcGGGGAAGGacCCCCCAAGTGG | 18 | X | No |
|  | OT27 | chr1 | 2570615 | 2571070 | chr1 | 2570837 | 2570860 | + | GTGGGGAAGGGcaCCCCtAGGGG | 17.39130435 | X | Yes |
|  | OT28 | chr1 | 2129135 | 2129360 | chr1 | 2129129 | 2129151 | - | GaGGGGAA-GaGCCCCCAgGAGG | 17.39130435 | RNA | Yes |
|  | OT29 | chr17 | 26583820 | 26584025 | chr17 | 26583814 | 26583837 | - | aTGGGGAtGGGaCCCCCAtGGGG | 16.66666667 | X | No |
|  | OT30 | chr1 | 23222242 | 23222483 | chr1 | 23222466 | 23222489 | + | GgGGGGAgGGacCtCCCAAGTGG | 16 | X | No |
|  | OT31 | chr1 | 2570615 | 2571070 | chr1 | 2570850 | 2570872 | - | GTGGGG-AGGcGtCCCCtAGGGG | 15.69565217 | RNA | No |
|  | OT32 | chr12 | 1.28E+08 | 1.28E+08 | chr12 | 127610178 | 127610201 | - | GTGGGGtAGaaaCCCCCtAGTGG | 15 | X | No |
|  | OT33 | chr10 | 52240528 | 52240791 | chr10 | 52240775 | 52240797 | + | cT-GGGAAGGaGCCCCCAtGGGG | 15 | RNA | Yes |
|  | OT34 | chrX | 16594536 | 16594797 | chrX | 16594530 | 16594553 | - | tTGGGGAAGGGGaCCCCAAcGGG | 14 | X | No |
|  | OT35 | chr1 | 2.27E+08 | 2.27E+08 | chr1 | 226885561 | 226885583 | + | GT-GatAAGGGGtCCCCAAGAGG | 12.12121212 | RNA | No |
|  | OT36 | chr11 | 45793335 | 45793605 | chr11 | 45793329 | 45793351 | - | GaGGGGAAGGGG-CCCCtAGAGG | 12.1 | RNA | No |
|  | OT37 | chr19 | 51703765 | 51703994 | chr19 | 51703759 | 51703781 | - | GT-GGGAgGGGcCtCCCAAGGGG | 11.84210526 | RNA | No |
|  | OT38 | chr9 | 1.37E+08 | 1.37E+08 | chr9 | 137040183 | 137040205 | + | GT-GaGAAGaGGtCCCCAAcAGG | 11.07692308 | RNA | No |
|  | OT39 | chr6 | 73977914 | 73978175 | chr6 | 73978158 | 73978181 | + | GTGtGGAAGGGGaCCCCAAcGGG | 10 | X | No |
|  | OT40 | chr5 | 1.18E+08 | 1.18E+08 | chr5 | 117819221 | 117819244 | - | GTGGGGtAaGaaCCCCCAAaTGG | 10 | X | No |
|  | OT41 | chr19 | 33399598 | 33399856 | chr19 | 33399592 | 33399614 | - | aT-GGGAAGGGGCtCCCtAGTGG | 9.6 | RNA | No |
|  | OT42 | chr17 | 73838834 | 73839136 | chr17 | 73838909 | 73838931 | - | aTGGGG-tGGGGtCCCCAAGGGG | 9.375 | RNA | Yes |
|  | OT43 | chr9 | 1.38E+08 | 1.38E+08 | chr9 | 138419293 | 138419315 | + | GTGGGG-AGcGGCCCCCcAGTGG | 9.257142857 | RNA | No |
|  | OT44 | chr17 | 79622772 | 79622999 | chr17 | 79622766 | 79622789 | - | GcaGGaAAGGGGaCCCCAAGAGG | 7.692307692 | X | Yes |
|  | OT45 | chr1 | 17030759 | 17030948 | chr1 | 17030753 | 17030776 | - | aTGGGGAAGaGaCCCCagAGAGG | 7.363636364 | X | No |
|  | OT46 | chr21 | 15464495 | 15464686 | chr21 | 15464669 | 15464691 | + | GTGtGGAAGGGGaCCCC-AGCGG | 7.111111111 | RNA | No |
|  | OT47 | chr10 | 1.18E+08 | 1.18E+08 | chr10 | 118153702 | 118153724 | - | GTGtGGAAGGGGaCCCC-AGTGG | 7 | RNA | No |
|  | OT48 | chr10 | 76584411 | 76584646 | chr10 | 76584606 | 76584628 | + | GgGGGGAA-GGGCCCCCcAGCGG | 6.666666667 | RNA | No |
|  | OT49 | chr1 | 1.49E+08 | 1.49E+08 | chr1 | 149282471 | 149282494 | + | ccGGGGAAaGGGCCCCCgAGGGG | 6.4 | X | No |
|  | OT50 | chr12 | 1.19E+08 | 1.19E+08 | chr12 | 118810037 | 118810059 | - | GaGGGGAAGGGt-CCCCgAGAGG | 6.25 | RNA | No |
|  | OT51 | chr1 | 2.11E+08 | 2.11E+08 | chr1 | 210862797 | 210862820 | + | aTGtGGggaGGGCaCCCAAGTGG | 6.25 | X | No |
|  | OT52 | chr3 | 52025380 | 52025613 | chr3 | 52025375 | 52025397 | - | GTGGGGAAaGGGaCCCC-AGTGG | 6.125 | RNA | No |
|  | OT53 | chr4 | 42658491 | 42658780 | chr4 | 42658690 | 42658712 | + | GgGGGGAAGGGt-CCCCtAGGGG | 5.882352941 | RNA | No |
|  | OT54 | chr14 | 95518855 | 95519200 | chr14 | 95518929 | 95518951 | - | G-GGGaAAGcaGCCCCCAgGGGG | 5.555555556 | RNA | No |
|  | OT55 | chr16 | 57286473 | 57286656 | chr16 | 57286467 | 57286490 | - | cTGGGGAAGGGcCCCCCAgGTGG | 5.444444444 | X | No |
|  | OT56 | chr1 | 59090101 | 59090386 | chr1 | 59090096 | 59090118 | - | aTGaGGAAGGGGaCCCC-AGCGG | 5.444444444 | RNA | No |
|  | OT57 | chr19 | 42435009 | 42435219 | chr19 | 42435203 | 42435225 | + | GTGGGG-AGGacCCCCCAAGGAG | 5.333333333 | RNA | No |

**Table S2. CROss-seq captured sites using *HEK2* gRNA and Cas9 *in vitro*.**

| **Site name** | **Peak Position** | | | **off-target site** | | | | **DNA sequence at off-target site** | **CROSS score** | **Bulge** | **Validated** |
| --- | --- | --- | --- | --- | --- | --- | --- | --- | --- | --- | --- |
|  | **chr** | **start** | **end** | **chr** | **start** | **end** | **strand** |  |  |  |  |
| HEK2 | chr5 | 87240196 | 87240613 | chr5 | 87240596 | 87240619 | + | GAACACAAAGCATAGACTGCGGG | 50 | X | No |
| OT1 | chrX | 30002771 | 30004349 | chrX | 30002761 | 30002784 | - | cAACAaAAttgATAGACcGCTAG | 4.166666667 | X | No |
| OT2 | chr18 | 24740750 | 24741319 | chr18 | 24740984 | 24741007 | - | cAACAaAAttgATAGACgGCTAG | 3 | X | No |

**Table S3. CROss-seq captured sites using *HBG* gRNA and Cas9 *in vivo*.**

| **Time** | **Site name** | **Peak Position** | | | **off-target site** | | | | **DNA sequence at off-target site** | **CROSS score** | **Bulge** | **Validated** |
| --- | --- | --- | --- | --- | --- | --- | --- | --- | --- | --- | --- | --- |
|  |  | **chr** | **start** | **end** | **chr** | **start** | **end** | **strand** |  |  |  |  |
| 24 hours | OT1 | chr1 | 149281999 | 149282819 | chr1 | 149282471 | 149282494 | + | ccGGGGAAaGGGCCCCCgAGGGG | 62.75871314 | X | No |
|  | OT2 | chr10 | 73282075 | 73282582 | chr10 | 73282203 | 73282225 | - | GTGGGG-AGtGGCCCCCAAGAGG | 38.71604938 | RNA | Yes |
|  | OT3 | chr16 | 67040163 | 67041266 | chr16 | 67040397 | 67040420 | - | GTGGcGAAGGGGCCCCCAAaGAG | 36.98 | X | No |
|  | OT4 | chr11 | 65163463 | 65164834 | chr11 | 65164126 | 65164149 | - | cTGGGtAAGGaGCCCaCAAGGGG | 36 | X | Yes |
|  | OT5 | chr10 | 95058670 | 95059189 | chr10 | 95059023 | 95059046 | + | GaGGGaAAGGaGCCCCCgAGGGG | 33 | X | Yes |
|  | OT6 | chr20 | 547509 | 548293 | chr20 | 548095 | 548118 | + | GTGGaGgAGaGGCCCCCAgGAGG | 30.86813187 | X | No |
|  | OT7 | chr17 | 79275938 | 79277148 | chr17 | 79276562 | 79276585 | - | acGGGGAAGaGaCCCCCAgGAGG | 25.97297297 | X | Yes |
|  | OT8 | chr17 | 48225258 | 48225746 | chr17 | 48225601 | 48225624 | + | GTGGGGAtGaGaCCCCCAAaAGG | 23.04 | X | No |
|  | OT9 | chr10 | 52240435 | 52240791 | chr10 | 52240775 | 52240797 | + | cT-GGGAAGGaGCCCCCAtGGGG | 22 | RNA | No |
|  | OT10 | chr2 | 73122104 | 73122814 | chr2 | 73122411 | 73122433 | - | cTGGGGgAGGaGCCCCC-AaGGG | 21 | RNA | No |
|  | HBG2 | chr11 | 5275915 | 5276219 | chr11 | 5276202 | 5276225 | + | GTGGGGAAGGGGCCCCCAAGAGG | 17 | X | Yes |
|  | HBG1 | chr11 | 5270991 | 5271295 | chr11 | 5271278 | 5271301 | + | GTGGGGAAGGGGCCCCCAAGAGG | 14 | X | Yes |
|  | OT11 | chr2 | 241421031 | 241421731 | chr2 | 241421714 | 241421738 | + | GcaGGGtgGGGGCCaCCACtGCAG | 13.06666667 | DNA | No |
|  | OT12 | chr3 | 38049978 | 38052410 | chr3 | 38051910 | 38051932 | + | GT-GtGAAGaGGaCaCCAgGTAG | 12 | RNA | No |
|  | OT13 | chr17 | 79622134 | 79623247 | chr17 | 79622766 | 79622789 | - | GcaGGaAAGGGGaCCCCAAGAGG | 10.88888889 | X | Yes |
|  | OT14 | chr17 | 27909791 | 27911015 | chr17 | 27910982 | 27911005 | + | GgGaGGAAGGGatCCCCAAGGGG | 10.5 | X | Yes |
|  | OT15 | chr1 | 230561330 | 230561967 | chr1 | 230561325 | 230561347 | - | GgcGGtAAGGGGCtgCC-cGCGG | 10.28571429 | RNA | No |
|  | OT16 | chr16 | 68344591 | 68345448 | chr16 | 68344888 | 68344912 | - | GCTGGGGggcGGGtCCtaAAGCAG | 9.090909091 | DNA | No |
|  | OT17 | chr15 | 75658233 | 75659180 | chr15 | 75658861 | 75658883 | - | G-GGGGAAaGaGaCCCCcgaAGG | 9 | RNA | No |
|  | OT18 | chr7 | 51095773 | 51096483 | chr7 | 51096411 | 51096433 | + | GTGGaG-gGGGGtCCCCtgGCAG | 8.066666667 | RNA | No |
|  | OT19 | chr2 | 8727287 | 8727832 | chr2 | 8727549 | 8727572 | - | GTGGaGgAGGGGCCCCCAcGTGG | 7.680851064 | X | No |
|  | OT20 | chr3 | 48686391 | 48687949 | chr3 | 48687379 | 48687401 | + | GTGGGG-AGGGGCCCCCAAGCAG | 7.5625 | RNA | No |
|  | OT21 | chr3 | 13705529 | 13706192 | chr3 | 13705831 | 13705853 | - | GT-GGGAtGGGGtCCCCAAGTGG | 7.534883721 | RNA | No |
|  | OT22 | chr22 | 30098700 | 30102296 | chr22 | 30099799 | 30099822 | + | aTGGGGAgGGGGaCaCCAgGGGG | 7.2 | X | No |
|  | OT23 | chr3 | 152552537 | 152553121 | chr3 | 152553104 | 152553128 | + | agGaGGAAGatGCCCCCAATtAAG | 7.142857143 | DNA | No |
|  | OT24 | chr1 | 53874168 | 53875116 | chr1 | 53874560 | 53874583 | + | GgGGGctAaGGGaCCCCAtGAGG | 7 | X | No |
|  | OT25 | chrX | 129325393 | 129326392 | chrX | 129325947 | 129325969 | - | tTGGGGgctGtGgCCCC-AGGAG | 6.125 | RNA | No |
|  | OT26 | chr20 | 34203887 | 34208233 | chr20 | 34206505 | 34206527 | - | GT-GGGAgGtGGgCgCCgtGGGG | 6.125 | RNA | No |
|  | OT27 | chr21 | 47674743 | 47676913 | chr21 | 47675045 | 47675067 | + | GTGGGcAA-atGCtgCCAgGTGG | 6 | RNA | No |
|  | OT28 | chr2 | 135725421 | 135726949 | chr2 | 135726933 | 135726955 | + | GTGGGGAgaGGG-agaCgAGAGG | 6 | RNA | No |
|  | OT29 | chr17 | 46653197 | 46654528 | chr17 | 46653866 | 46653888 | - | cacGGGgA-GGcCCCCCcAGAAG | 6 | RNA | No |
|  | OT30 | chr3 | 133545831 | 133548875 | chr3 | 133546951 | 133546973 | - | GcccGGcAGcaGCCCCC-AGAGG | 5.555555556 | RNA | No |
|  | OT31 | chr19 | 35984480 | 35987685 | chr19 | 35986524 | 35986546 | + | GgaGaGAAGGGa-ggCCAAGCAG | 5.4 | RNA | No |
|  | OT32 | chr22 | 37465716 | 37467282 | chr22 | 37466895 | 37466919 | - | GgaGGGAgGGGGtgCCCACAGCAG | 5 | DNA | No |
|  | OT33 | chr20 | 61402095 | 61403171 | chr20 | 61402089 | 61402111 | - | GTGaGG-cGGGGCCCtCcctGGG | 5 | RNA | No |
|  | OT34 | chr19 | 50117640 | 50119571 | chr19 | 50119242 | 50119264 | + | G-GGGtgAGcGGgCCaCcAGCGG | 5 | RNA | No |
|  | OT35 | chr16 | 89330613 | 89333826 | chr16 | 89331198 | 89331222 | - | cTaGaGAGAGGGaCCCaCAgGCAG | 5 | DNA | No |
|  | OT36 | chr15 | 41952146 | 41952844 | chr15 | 41952445 | 41952467 | - | tT-GGGAcccGcCCCCCgAGTGG | 5 | RNA | No |
| 12 hours | OT1 | chr10 | 73282132 | 73282716 | chr10 | 73282203 | 73282225 | - | GTGGGG-AGtGGCCCCCAAGAGG | 18.2826087 | RNA | Yes |
|  | HBG2 | chr11 | 5275908 | 5276219 | chr11 | 5276202 | 5276225 | + | GTGGGGAAGGGGCCCCCAAGAGG | 12 | X | Yes |
|  | OT2 | chr19 | 41118910 | 41119956 | chr19 | 41119498 | 41119520 | + | G-GGaGAAGaGGgCgaaAAGGGG | 11 | RNA | No |
|  | OT3 | chr16 | 71972086 | 71972883 | chr16 | 71972081 | 71972103 | - | GTGGGGAgaGGcCatgC-AGTGG | 9.090909091 | RNA | No |

**Table S4. CROss-seq captured sites using *HEK2* gRNA and Cas9 *in vivo*.**

| **Site name** | **Peak Position** | | | **off-target site** | | | | **DNA sequence at off-target site** | **CROSS score** | **Bulge** | **Validated** |
| --- | --- | --- | --- | --- | --- | --- | --- | --- | --- | --- | --- |
|  | **chr** | **start** | **end** | **chr** | **start** | **end** | **strand** |  |  |  |  |
| HEK2 | chr5 | 87240294 | 87240613 | chr5 | 87240596 | 87240619 | + | GAACACAAAGCATAGACTGCGGG | 103 | X | No |
| OT1 | chr13 | 113177376 | 113177766 | chr13 | 113177581 | 113177605 | + | GAAgACtAAGCAagGACAaGaAAG | 5.333333333 | DNA | No |

**Table S5. CROss-seq captured sites using *HEK2* gRNA and BE3 *in vitro*.**

| **Site name** | **Peak Position** | | | **off-target site** | | | | **DNA sequence at off-target site** | **CROSS score** | **Bulge** | **Validated** |
| --- | --- | --- | --- | --- | --- | --- | --- | --- | --- | --- | --- |
|  | **chr** | **start** | **end** | **chr** | **start** | **end** | **strand** |  |  |  |  |
| HEK2 | chr5 | 87240257 | 87240768 | chr5 | 87240596 | 87240619 | + | GAACACAAAGCATAGACTGCGGG | 16 | X | Yes |
| OT1 | chr6 | 144610327 | 144610667 | chr6 | 144610321 | 144610345 | - | GCAACACtgAGgATAGcagGCTGG | 4.166666667 | DNA | No |
| OT2 | chr3 | 6788585 | 6789287 | chr3 | 6788579 | 6788603 | - | tAACgCAACAGgAgAGttTGCCAG | 4.166666667 | DNA | No |
| OT3 | chr4 | 121884509 | 121884876 | chr4 | 121884769 | 121884791 | + | GAAgACgtgGtATc-ACTGCAGG | 3.571428571 | RNA | No |
| OT4 | chr11 | 121237130 | 121237395 | chr11 | 121237379 | 121237401 | + | cAACAaAAcaCATAcA-gGCAAG | 3.571428571 | RNA | No |
| OT5 | chr8 | 103892087 | 103892550 | chr8 | 103892126 | 103892148 | - | GtACtCAgAaCATgG-CaGCTGG | 3.2 | RNA | No |
| OT6 | chr5 | 142216946 | 142217231 | chr5 | 142216941 | 142216963 | - | GAACACAAAGCActaAg-GCTGG | 3.2 | RNA | No |
| OT7 | chr1 | 94574398 | 94575199 | chr1 | 94574802 | 94574826 | - | acACACAgAGCATGgGACcGaGAG | 3.2 | DNA | No |
| OT8 | chr11 | 94341495 | 94341983 | chr11 | 94341624 | 94341646 | + | ttACACAtAGCtTA-ACTttAAG | 3.2 | RNA | No |
| OT9 | chr12 | 102256530 | 102257856 | chr12 | 102257014 | 102257036 | + | GgtCAC-AtGaATAGAtTGaAGG | 3.125 | RNA | No |
| OT10 | chr6 | 106579966 | 106580485 | chr6 | 106580311 | 106580333 | + | GAcCACAAAGgcTc-AtTGaAAG | 3 | RNA | No |
| OT11 | chr5 | 114737859 | 114738188 | chr5 | 114738173 | 114738196 | + | GgACACAgAGatTtGtCTGCTGG | 3 | X | No |

**Table S6. CROss-seq captured sites using *HEK2* gRNA and BE4max *in vivo*.**

| **Site name** | **Peak Position** | | | **off-target site** | | | | **DNA sequence at off-target site** | **CROSS score** | **Bulge** | **Validated** |
| --- | --- | --- | --- | --- | --- | --- | --- | --- | --- | --- | --- |
|  | **chr** | **start** | **end** | **chr** | **start** | **end** | **strand** |  |  |  |  |
| HEK2 | chr5 | 87240494 | 87240730 | chr5 | 87240596 | 87240619 | + | GAACACAAAGCATAGACTGCGGG | 16 | X | Yes |

**Table S7. CROss-seq captured sites using *HEK2* gRNA and ABE7.10 *in vitro*.**

| **Site name** | **Peak Position** | | | **off-target site** | | | | **DNA sequence at off-target site** | **CROSS score** | **Bulge** | **Validated** |
| --- | --- | --- | --- | --- | --- | --- | --- | --- | --- | --- | --- |
|  | **chr** | **start** | **end** | **chr** | **start** | **end** | **strand** |  |  |  |  |
| HEK2 | chr5 | 87240407 | 87240613 | chr5 | 87240596 | 87240619 | + | GAACACAAAGCATAGACTGCGGG | 21.33333333 | X | Yes |
| OT1 | chr4 | 175335466 | 175336163 | chr4 | 175335957 | 175335980 | - | cAACAaAAttgATAGACcGCTAG | 6 | X | No |
| OT2 | chr1 | 180455140 | 180456036 | chr1 | 180455536 | 180455560 | - | tggCAgAAAGCATAAGACTctTGG | 6 | DNA | No |
| OT3 | chr7 | 92437472 | 92437960 | chr7 | 92437769 | 92437791 | + | GAA-ACAAAtgATtcACTtaAAG | 5 | RNA | No |
| OT4 | chr6 | 37386411 | 37386949 | chr6 | 37386718 | 37386740 | - | cAAtA-AAAGCATAaAgTGaAGG | 4.5 | RNA | No |
| OT5 | chrX | 83125155 | 83125403 | chrX | 83125231 | 83125253 | + | GAAggCAAA-tATAGtCaGaGAG | 4.166666667 | RNA | No |
| OT6 | chr3 | 162220905 | 162221300 | chr3 | 162221282 | 162221306 | + | GAACAtAtAtCAaAGTACTaaGAG | 4.166666667 | DNA | No |
| OT7 | chr7 | 129857725 | 129858509 | chr7 | 129857870 | 129857892 | + | GAAaACAAgGCgcAGAt-cCCAG | 4 | RNA | No |
| OT8 | chr20 | 49796635 | 49797109 | chr20 | 49796747 | 49796769 | - | GAACAaAtA-CAgAagCTaCAGG | 4 | RNA | No |
| OT9 | chr11 | 47494992 | 47495174 | chr11 | 47495071 | 47495095 | + | GAgCAgGAAAaCAgAGACTGtAGG | 3.6 | DNA | No |
| OT10 | chr2 | 236674274 | 236674498 | chr2 | 236674443 | 236674465 | + | GAACACcAA-CATAGtCatCTGG | 3.571428571 | RNA | No |
| OT11 | chr19 | 44646114 | 44646365 | chr19 | 44646109 | 44646131 | - | GAACAgcAAGgAgAGgC-cCAGG | 3.571428571 | RNA | No |
| OT12 | chr11 | 59315185 | 59315495 | chr11 | 59315307 | 59315329 | - | GAcCAtAAAGg-TgGACaGCCAG | 3.571428571 | RNA | No |
| OT13 | chr10 | 60410873 | 60411338 | chr10 | 60410868 | 60410890 | - | GgtCACAAAGCtTAGAg-cgTGG | 3.571428571 | RNA | No |
| OT14 | chr6 | 1586490 | 1586673 | chr6 | 1586484 | 1586506 | - | GAgCAacAAtCATA-ACTaaTAG | 3.2 | RNA | No |
| OT15 | chr2 | 96944175 | 96944501 | chr2 | 96944485 | 96944507 | + | G-AtgCccAGCAaAGACTGtGAG | 3.2 | RNA | No |
| OT16 | chr22 | 22344674 | 22345076 | chr22 | 22344701 | 22344725 | - | acACACAggGCCATAGAaaGCCAG | 3.2 | DNA | No |
| OT17 | chr14 | 74828712 | 74828980 | chr14 | 74828694 | 74828716 | - | aAtCcCAAAGgAaA-ACaGCAGG | 3.2 | RNA | No |
| OT18 | chr12 | 39279698 | 39279932 | chr12 | 39279916 | 39279938 | + | GcAC-CcAAGaAgAGACTGCCAG | 3.2 | RNA | No |
| OT19 | chr11 | 66097065 | 66097360 | chr11 | 66097058 | 66097082 | - | GAAaACAAAaaAgAaACTGAtTGG | 3.2 | DNA | No |
| OT20 | chr14 | 103634699 | 103634932 | chr14 | 103634696 | 103634718 | - | aAAtgCAAA-CAaAtACTGaAAG | 3.125 | RNA | No |
| OT21 | chr12 | 12016685 | 12017285 | chr12 | 12017214 | 12017236 | + | GAcatCAAAG-AaAGcCTGCAGG | 3.125 | RNA | No |
| OT22 | chr7 | 38841692 | 38842063 | chr7 | 38841906 | 38841928 | - | tAACAtcAAGgAgAGA-aGCAGG | 3 | RNA | No |
| OT23 | chr5 | 112204937 | 112205150 | chr5 | 112204920 | 112204944 | - | GAACAatAAtCATAaAgGTGtTAG | 3 | DNA | No |
| OT24 | chr3 | 19329069 | 19329304 | chr3 | 19329049 | 19329073 | - | cAACAACAAAaagTgGACTGtGGG | 3 | DNA | No |
| OT25 | chr20 | 24473008 | 24473393 | chr20 | 24472988 | 24473012 | - | GAACCAgAcAGCAgAGgCgGCGGG | 3 | DNA | No |

**Table S8. CROss-seq captured sites using *HEK2* gRNA and ABE8e *in vivo*.**

| **Site name** | **Peak Position** | | | **off-target site** | | | | **DNA sequence at off-target site** | **CROSS score** | **Bulge** | **Validated** |
| --- | --- | --- | --- | --- | --- | --- | --- | --- | --- | --- | --- |
|  | **chr** | **start** | **end** | **chr** | **start** | **end** | **strand** |  |  |  |  |
| HEK2 | chr5 | 87240417 | 87240669 | chr5 | 87240596 | 87240619 | + | GAACACAAAGCATAGACTGCGGG | 8.33333 | X | Yes |
| OT1 | chr1 | 100075693 | 100076021 | chr1 | 100075853 | 100075877 | + | aAAtAtTAcAGCATAGACTGCAGG | 2.57143 | DNA | No |
| OT2 | chr18 | 5596013 | 5596571 | chr18 | 5596259 | 5596281 | - | ctACAg-AAGCATAGACTGCAGG | 2.08333 | RNA | No |
| OT3 | chr10 | 127539781 | 127540374 | chr10 | 127540181 | 127540203 | + | cAcCcCAAAatAT-GACTGgAGG | 1.33333 | RNA | No |
| OT4 | chr6 | 139352806 | 139353270 | chr6 | 139353001 | 139353023 | + | cAA-ACAAAaCATAGACTGCTGG | 0.925926 | RNA | No |
| OT5 | chr2 | 75812857 | 75813185 | chr2 | 75813026 | 75813050 | + | aAAggaAcAcTCATAGACTGCTGG | 0.8 | DNA | No |
| OT6 | chr5 | 140366811 | 140367150 | chr5 | 140366851 | 140366873 | - | t-tgttAAAaCATAGACTGCCGG | 0.571429 | RNA | No |

**Table S9. CROss-seq captured sites using *HEK3* pegRNA and PE2 *in vivo*.**

| **Site name** | **Peak Position** | | | **off-target site** | | | | **DNA sequence at off-target site** | **CROSS score** | **Bulge** | **Validated** |
| --- | --- | --- | --- | --- | --- | --- | --- | --- | --- | --- | --- |
|  | **chr** | **start** | **end** | **chr** | **start** | **end** | **strand** |  |  |  |  |
| HEK3 | chr9 | 110184384 | 110185017 | chr9 | 110184619 | 110184642 | + | GGCCCAGACTGAGCACGTGATGG | 8.75676 | X | Yes |

**Table S10. CROss-seq captured sites using *HEK4* pegRNA and PE2 *in vivo*.**

| **Site name** | **Peak Position** | | | **off-target site** | | | | **DNA sequence at off-target site** | **CROSS score** | **Bulge** | **Validated** |
| --- | --- | --- | --- | --- | --- | --- | --- | --- | --- | --- | --- |
|  | **chr** | **start** | **end** | **chr** | **start** | **end** | **strand** |  |  |  |  |
| HEK4 | chr20 | 31349319 | 31349819 | chr20 | 31349755 | 31349778 | + | GGCACTGCGGCTGGAGGTGGGGG | 2 | X | Yes |
| OT1 | chr10 | 126694562 | 126695565 | chr10 | 126694857 | 126694880 | + | GGCACgaCGGCTGGAGGTGGGGG | 1 | X | Yes |

**Table S11. Sequences of sgRNAs and pegRNAs used in this study.**

| **sgRNA** | **Sequence** |  |  |  |
| --- | --- | --- | --- | --- |
| HBG | GTGGGGAAGGGGCCCCCAAGagg |  |  |  |
| HEK2 | GAACACAAAGCATAGACTGCggg |  |  |  |
| HEK4 | GGCACTGCGGCTGGAGGTGGggg |  |  |  |
| FANCF | GGAATCCCTTCTGCAGCACCtgg |  |  |  |
|  |  |  |  |  |
| **pegRNA** | **spacer sequence** | **3' extension** | **PBS length (nt)** | **RT template length (nt)** |
| HEK3 (+1CTTins) | GGCCCAGACTGAGCACGTGA | TCTGCCATCAAAGCGTGCTCAGTCTG | 13 | 10 |
| HEK4 (+3TAAins) | GGCACTGCGGCTGGAGGTGG | TTAACCCCTTACACCTCCAGCC | 9 | 13 |

**Table S12. Sequence of primers used for deep sequencing of off-target sites captured by *HBG* gRNA and Cas9 *in vitro*.**

| **Site name** | **Forward (5' to 3')** | **Reverse (5' to 3')** | **Barcode of treat sample** | | **Barcode of control sample** | |
| --- | --- | --- | --- | --- | --- | --- |
|  |  |  | **Forward** | **Reverse** | **Forward** | **Reverse** |
| HBG1 | ccatgggtggagtttagcca | gactgaatcggaacaaggca | AATCCGGA | CTACAGTT | CAACAATG | CTTCACGG |
| HBG2 | gtggagtttagccagggaccg | agtcctggtatcttctatggtgg | TAATACAG | ATATTCAC | TGGTGGCA | TCCTGTAA |
| OT1 | acagcccccatatgacatga | atgtgaagtcagggttgagc | ATCCACTG | AGGTGCGT | GCGCAAGC | TCACGCCG |
| OT2 | gtggaagcccaggtcaaaca | tcccttcgccatcatctgtc | TTATAACC | GATATCGA | TAAGTGGT | CTTAAGCC |
| OT3 | ctcaccctgggctttctcag | ctgggctggggagagaaaag | TCCAACGC | TTGGACTT | ACACTAAG | ATCCATAT |
| OT4 | cccagccaggattggctttc | gagatgagagggccaaagccc | GCTTGTCA | GAACATAC | AAGATACT | ACTTACAT |
| OT5 | acacccgtcacagctagaaa | atgtctgtggaacggtaagaca | AATGCCTC | TCGATCCA | GCCACAGG | CATGCCAT |
| OT6 | cacacatagtgcctggtggt | ctgaaggactttggggtgct | CGGCGTGA | GCGCCTGT | AGGCAGAG | AGAATGCC |
| OT7 | gccggcagatgtcatagggg | caaggtggcacgctgtactc | GGCTTAAG | TCGTGACC | GGTGAACC | GCGTTGGA |
| OT8 | agatgatgtccgtgtcaacct | ctgatccgtgtcctccacct | GGCATTCT | CTAGCTTG | CCTTCACC | GACGCTCC |
| OT9 | gtggagtgtcagagaggtgg | caacatccctatgtgccagg | GGACTTGG | CGCAGACG | CGGACAAC | TCCGGATT |
| OT10 | gccactgctaccagtctctc | gacgggtgtgatctgtcagg | TCTCTACT | CGCGGTTC | ACCTTGGC | GGCCTCAT |
| OT11 | ggcatctccatcttgcaggt | ggggaaggagtaccattggc | CCGCGGTT | AGCGCTAG | CTACGACA | GAGTCCAA |
| OT12 | gctctgccttctcacctcag | gcctaagttcgggggaaagg | GGTACCTT | AAGACGTC | CATAATAC | TTCTAACG |
| OT13 | aatctctgtccagccactgc | actgcctgaggtcacgaaag | TTACAGGA | TGACAAGC | TTCCTGTT | AGTATCTT |
| OT15 | aggtgccttggcttgctatt | ctgggaggagagttggggta | CTCTCGTC | TATAACCT | ATATCTCG | ATCTTAGT |
| OT16 | cgcaccaagacagcagttcg | ccgggagtatcagtccctcc | ATGAGGCC | GTTAATTG | CGGAACTG | CACTACGA |
| OT17 | cttgtgtcagcaccacatgc | taacatgtaagcccctggga | GCACGGAC | GTCTCGCA | TGCGGCGT | CCTCGGTA |
| OT19 | gaggtgcaagaattggggga | tggcaagggtcagtgtcttc | GCAGAATT | ACCGGCCA | AGCTCGCT | GCAGAATC |
| OT20 | attgctgtactcacgtggcag | agccctataatggggaaggtcc | CCGTGAAG | CAGTGGAT | GTGTCGGA | GCTTGCGC |
| OT23 | aagagctagcactgcacctg | gccagtggtgttgggttttc | CTTGGTAT | CCAAGTCC | CCATTCGA | GTTGTCCG |
| OT27 | gggctggcatctacactcag | gagcctggtcacagcagaag | CGTTAGAA | TTCAGGTC | ACTCGTGT | ATTGGAAC |
| OT28 | gacatgggaggaggggagat | atttacccttggccaccagg | AGCCTCAT | AGTAGAGA | GTCTACAC | GCCAAGGT |
| OT30 | ggagtttagtgggtggtgga | agaagtcaaggaactggccc | GATTCTGC | GACGAGAG | CAATTAAC | CGAGATAT |
| OT32 | tgtgtgtgtctccatgaccg | ttaccaggacaggtgagggt | TTGGACTC | GGAAGCAG | AACAGGTT | ATACCAAG |
| OT33 | aatttcagcaaccccagccag | ttggtccagactgctcggact | ATGTAAGT | ACTCTATG | GAATGAGA | GAGGCATT |
| OT37 | tgtgcaaagagactcccagg | gtggtcccagtcctacctct | AAGTCCAA | TATGAGTA | ATATGGAT | CTGTATTA |
| OT38 | gcttgctggtctgagaggtc | agctggttcttccacccaac | AACGTTCC | GGAGTACT | GATCTATC | ATGAGGCT |
| OT39 | cccaaagagtaagcagcagcaa | tcagcgccctgtccaaaacag | ACTAAGAT | AACCGCGG | TAAGGTCA | TGTCGTAG |
| OT40 | ggccctcatggagaacctctgc | gccaacagtgcaagctgtca | GTCGGAGC | GGTTATAA | TTGCCTAG | ACCACTTA |
| OT41 | cctttcccaaactcctcccc | ggacaccttggagctttgga | CAAGCTAG | ACATAGCG | GGAGCGTC | GTCCGTGC |
| OT42 | aaacaggccaagggcattct | cgctacagggcttcttggaa | GACCTGAA | TTGGTGAG | GTTCCAAT | AATTCTGC |
| OT44 | gagtcaggaatcccagccaa | gccggaaagttctggaagga | AGTTCAGG | CCAACAGA | GCAATGCA | GGAACGTT |
| OT54 | ggggttttacacaccccaca | ttgaagacaccccttcctgg | CCAAGTCT | AAGGATGA | GCGCTCTA | GCTCCGAC |

**Table S13. Sequence of primers used for deep sequencing of off-target sites captured by *HBG* gRNA and Cas9 *in vivo*.**

| **Site name** | **Forward (5' to 3')** | **Reverse (5' to 3')** | **Barcode of treat sample** | | **Barcode of control sample** | |
| --- | --- | --- | --- | --- | --- | --- |
|  |  |  | **Forward** | **Reverse** | **Forward** | **Reverse** |
| HBG2 | GTGGAGTTTAGCCAGGGACC | TGGTGGGAGAAGAAAACTAGC | CTACAGTT | AATCCGGA | AGGTGCGT | ATCCACTG |
| OT2 | agtgacacacagatgtaagtggt | cttaaaggtagccactgtcctgg | GGCTTAAG | TCGTGACC | AAGTCCAA | TATGAGTA |
| OT4 | GTGGAGGAAGGAAGACAGGTG | TACAGCTGCTCTCGGCAGAA | TAATACAG | ATATTCAC | GCTTGTCA | GAACATAC |
| OT5 | gCagacccataaaacgatgct | CCTGGGTCACTGGGATAAACAAA | TTGGACTC | GGAAGCAG | GGACTTGG | CGCAGACG |
| OT7 | GCGTTAAGAAGGGCTATGGATCT | TCTAGGAACTCTGGTTCCCATCT | GCACGGAC | GTCTCGCA | AGTTCAGG | CCAACAGA |
| OT8 | AGGCTGAATGTGCTAGCCAAG | GTAGTGGGCATGCCTTTACTCTT | ATGTAAGT | ACTCTATG | TGGATCGA | GTGCGATA |
| OT13 | CCCTTGCTTTCTGATCACTCTCA | GTTCAGCTTCCCCTAAGCCTATT | GGTACCTT | AAGACGTC | GACCTGAA | TTGGTGAG |
| OT14 | TCCCAGGAAACGAGGTCTCT | AGGCTCCAGCATGAAAGGAC | CGGCGTGA | GCGCCTGT | CAAGCTAG | ACATAGCG |
| OT285 | AGTGTGGAGCCCACTCCTTG | TGAGCTCCAGCATTAGACAAAGG | CCAAGTCT | AAGGATGA | TTATAACC | GATATCGA |
| OT393 | GGCTTAGACCTCTCCCTTATGTG | GACTGTCACTATGGTGggacaaa | CTCTCGTC | TATAACCT | CCGCGGTT | AGCGCTAG |

**REFERENCES**

Amemiya, H.M., Kundaje, A., and Boyle, A.P.(2019). The ENCODE blacklist: identification of problematic regions of the genome. SCI REP-UK 9, 1-5.

Bae, S., Park, J., and Kim, J.(2014). Cas-OFFinder: a fast and versatile algorithm that searches for potential off-target sites of Cas9 RNA-guided endonucleases. BIOINFORMATICS 30, 1473-1475.

Clement, K., Rees, H., Canver, M.C., Gehrke, J.M., Farouni, R., Hsu, J.Y., Cole, M.A., Liu, D.R., Joung, J.K., and Bauer, D.E.(2019). CRISPResso2 provides accurate and rapid genome editing sequence analysis. NAT BIOTECHNOL 37, 224-226.

Langmead, B., and Salzberg, S.L.(2012). Fast gapped-read alignment with Bowtie 2. NAT METHODS 9, 357.

Li, H., Handsaker, B., Wysoker, A., Fennell, T., Ruan, J., Homer, N., Marth, G., Abecasis, G., and Durbin, R.(2009). The sequence alignment/map format and SAMtools. BIOINFORMATICS 25, 2078-2079.

Liang, P., Xie, X., Zhi, S., Sun, H., Zhang, X., Chen, Y., Chen, Y., Xiong, Y., Ma, W., and Liu, D.(2019). Genome-wide profiling of adenine base editor specificity by EndoV-seq. NAT COMMUN 10, 1-9.

Martin, M.(2011). Cutadapt removes adapter sequences from high-throughput sequencing reads. EMBnet. journal 17, 10-12.

McKenna, A., Hanna, M., Banks, E., Sivachenko, A., Cibulskis, K., Kernytsky, A., Garimella, K., Altshuler, D., Gabriel, S., and Daly, M.(2010). The Genome Analysis Toolkit: a MapReduce framework for analyzing next-generation DNA sequencing data. GENOME RES 20, 1297-1303.

Quinlan, A.R., and Hall, I.M.(2010). BEDTools: a flexible suite of utilities for comparing genomic features. BIOINFORMATICS 26, 841-842.

Thorvaldsdóttir, H., Robinson, J.T., and Mesirov, J.P.(2013). Integrative Genomics Viewer (IGV): high-performance genomics data visualization and exploration. BRIEF BIOINFORM 14, 178-192.

Waterhouse, A.M., Procter, J.B., Martin, D.M., Clamp, M., and Barton, G.J.(2009). Jalview Version 2—a multiple sequence alignment editor and analysis workbench. BIOINFORMATICS 25, 1189-1191.

Zhang, Y., Liu, T., Meyer, C.A., Eeckhoute, J., Johnson, D.S., Bernstein, B.E., Nusbaum, C., Myers, R.M., Brown, M., and Li, W.(2008). Model-based analysis of ChIP-Seq (MACS). GENOME BIOL 9, 1-9.
